# Genome-wide association study for kernel composition and flour pasting behavior in wholemeal maize flour

**DOI:** 10.1186/s12870-019-1729-7

**Published:** 2019-04-02

**Authors:** Mara Lisa Alves, Bruna Carbas, Daniel Gaspar, Manuel Paulo, Cláudia Brites, Pedro Mendes-Moreira, Carla Moita Brites, Marcos Malosetti, Fred van Eeuwijk, Maria Carlota Vaz Patto

**Affiliations:** 1 Instituto de Tecnologia Química e Biológica António Xavier, Universidade Nova de Lisboa, Oeiras, Portugal; 2 Instituto Nacional de Investigação Agrária e Veterinária, Oeiras, Portugal; 3 Instituto Politécnico de Coimbra - Escola Superior Agrária, Coimbra, Portugal; 4 Wageningen University & Research, Wageningen, The Netherlands

**Keywords:** *Zea mays* L., Nutritional quality, Pasting behavior, Portuguese maize germplasm, Bread, Candidate genes, Plant breeding

## Abstract

**Background:**

Maize is a crop in high demand for food purposes and consumers worldwide are increasingly concerned with food quality. However, breeding for improved quality is a complex task and therefore developing tools to select for better quality products is of great importance. Kernel composition, flour pasting behavior, and flour particle size have been previously identified as crucial for maize-based food quality. In this work we carried out a genome-wide association study to identify genomic regions controlling compositional and pasting properties of maize wholemeal flour.

**Results:**

A collection of 132 diverse inbred lines, with a considerable representation of the food used Portuguese unique germplasm, was trialed during two seasons, and harvested samples characterized for main compositional traits, flour pasting parameters and mean particle size. The collection was genotyped with the MaizeSNP50 array. SNP-trait associations were tested using a mixed linear model accounting for genetic relatedness. Fifty-seven genomic regions were identified, associated with the 11 different quality-related traits evaluated. Regions controlling multiple traits were detected and potential candidate genes identified. As an example, for two viscosity parameters that reflect the capacity of the starch to absorb water and swell, the strongest common associated region was located near the *dull endosperm 1* gene that encodes a starch synthase and is determinant on the starch endosperm structure in maize.

**Conclusions:**

This study allowed for identifying relevant regions on the maize genome affecting maize kernel composition and flour pasting behavior, candidate genes for the majority of the quality-associated genomic regions, or the most promising target regions to develop molecular tools to increase efficacy and efficiency of quality traits selection (such as “breadability”) within maize breeding programs.

**Electronic supplementary material:**

The online version of this article (10.1186/s12870-019-1729-7) contains supplementary material, which is available to authorized users.

## Background

Maize is one of the most important food crops worldwide, and a staple crop for large populations in Latin America, Africa, and Asia [1]. Maize breeding has primarily focused on increasing stability and grain yield potential. However, in the last decade, much effort has also been made in improving maize for animal feed and human nutrition [2]. Maize food quality depends on the raw material composition and the way that raw material is processed, which varies greatly from country to country. For instance, in Spain or Portugal, wholemeal maize flour is still frequently used for ethnic maize leavened bread production for which the local maize populations are usually preferred [3]. When using maize kernel for baking purposes, the improvement of the end-product quality can be achieved by taking into consideration the upstream processes (e.g., harvest procedures, seed quality, pest control), but also the downstream processes (e.g., milling type, baking procedure). Central to this improvement is the cultivated maize genotype, and directly related to this is its kernel composition and associated technological ability for bread production (“breadability”). A better understanding of the complex genetic basis of maize kernel main components and technological/processing traits is essential for devising more efficient breeding tools to support the improvement of this crop compositional quality.

The maize kernel’s high nutritional value is mainly due to its starch (~ 72% of the kernel dry matter), protein (~ 10% of the kernel dry matter), and fat in the form of oil (~ 4% of the kernel dry matter) [4, 5]. The interactions of these three major components and fiber in a cereal-based food system are important to functionality and quality [6–8].

Many maize mutants have been widely used to isolate genes encoding key enzymes in starch metabolism, as well as genes regulating zeins, maize primary storage proteins, synthesis and deposition [9, 10]. Currently, high-throughput genomics and post-genomics approaches are providing new tools to better understand the genetic and biochemical networks operating during maize kernel development, contributing ultimately to its composition, and a high degree of complexity and regulation has been found [9].

Linkage mapping and association mapping studies have shown that kernel main components are controlled by many genes having complex patterns of inheritance. Examples are the works of Li et al. [11] and Li et al. [12] that carried out genome-wide association studies (GWAS) to study the genetic architecture of oil and amylose biosynthesis in maize kernels, respectively. Also the work conducted by Cook et al. [13] is to be mentioned, that used a joint-linkage mapping/GWAS approach to study the genetic architecture of kernel starch, protein, and oil content. Despite the importance of maize for food, fewer works have evaluated and identified genomic regions controlling pasting properties, contrary to what has already been described in rice [14, 15]. Wilson et al. [14], using a candidate gene approach with maize genes from the starch biosynthesis pathway, identified haplotypes of *brittle endosperm2* (*bt2*), *shrunken1* (*sh1*), and *shrunken2* (*sh2*) that were associated with several kernel compositional traits, and haplotypes of *amylose extender1* (*ae1*) and *sh2* that were associated with starch pasting properties.

The Portuguese maize germplasm is recognized for its high diversity [16, 17] and associated potential quality for food, in particular for the production of the ethnic leavened maize-based *broa* [17], that might be associated with their higher protein contents and lower breakdown viscosities values than the values found in commercial maize varieties [18]. However this national diversity was never properly exploited for the development of efficient tools/innovative approaches to support breeding for these complex quality traits, such as “breadability”.

The present study was carried out to identify genomic regions controlling the variation of maize kernel major constituents (protein, fiber, fat, and starch content) and parameters affecting the maize flour “breadability” (starch pasting properties and flour’s mean particle size) through a genome-wide association approach, using a unique association panel constituted by a collection of maize inbred lines in which a considerable amount of the unexplored Portuguese maize germplasm is present.

This will allow for the understanding of the genetic architecture of quality traits, the identification of candidate quality genes, and the development of molecular selection tools for quality-related traits difficult to select by conventional methods.

## Results

Portuguese traditional maize varieties known for their high “breadability” are overall characterized by lower viscosities and higher protein content than the commercial maize varieties. The present study was carried out to identify genomic regions controlling the variation of maize kernel major constituents (protein, fiber, fat, and starch content) and parameters affecting the maize flour “breadability” (starch pasting properties and flour’s mean particle size) through a genome-wide association approach. For that a collection of 132 maize inbred lines, with a significant representation of lines selected from Portuguese traditional maize populations (obtained by single seed descent), was trialed during two growing seasons. The collection lines were genotyped with the MaizeSNP50 BeadChip array and the samples harvested from each field replicate characterized for main compositional traits (protein, fiber, fat, and starch), flour pasting parameters (viscosity profiles), and mean particle size.

### Phenotypic variation of maize flour compositional and pasting properties traits

**Fig. 1:**
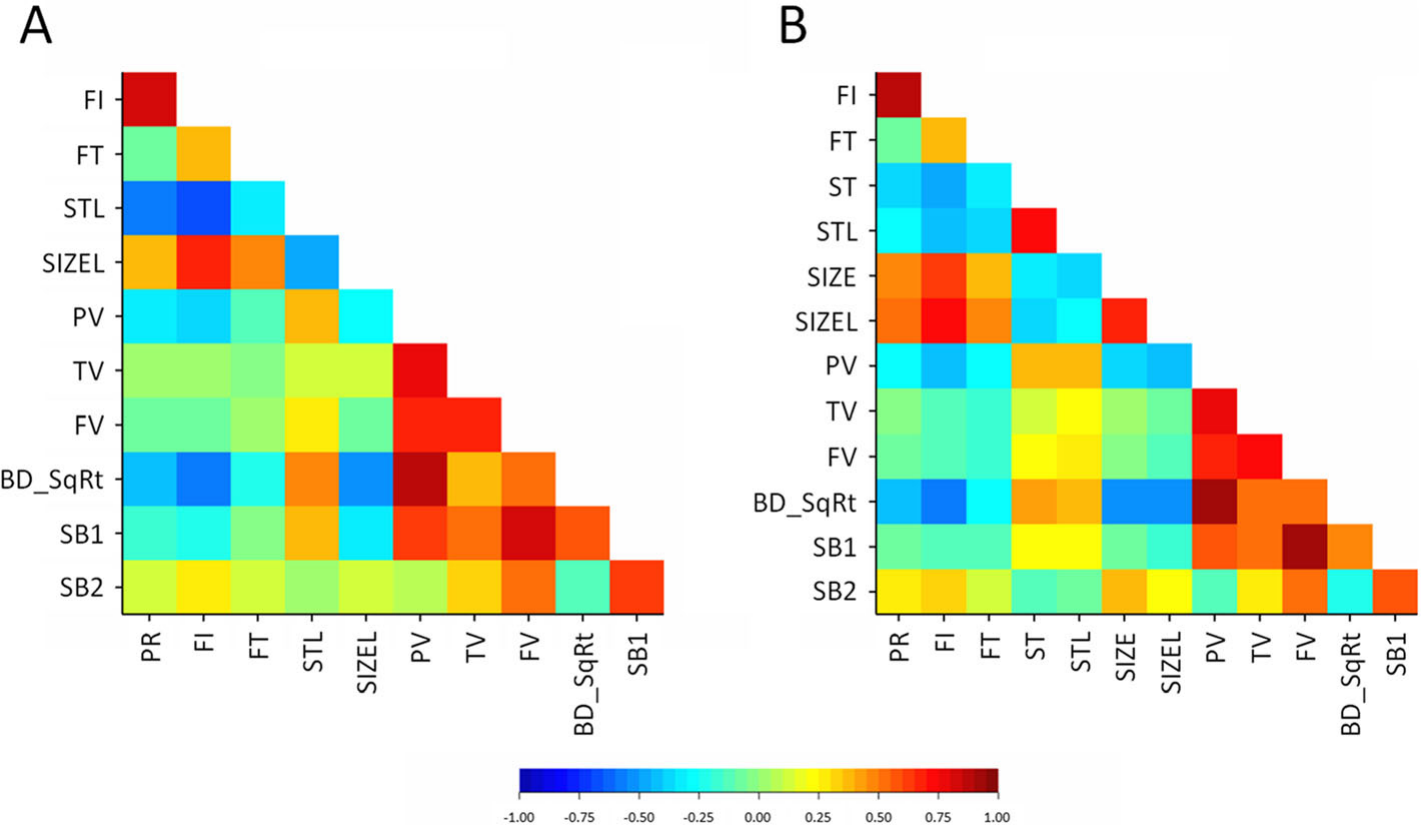
Heatmaps of genetic correlations for 11 quality traits measured in 132 maize inbred lines. **a** 2011 growing season, **b** 2012 growing season. Quality traits: PR – Protein content, FI – Fiber content, FT – Fat content, ST – Starch content in non-lyophilized flour, STL – Starch content in lyophilized flour, SIZE – Mean particle size in non-lyophilized flour, SIZEL – Mean particle size in lyophilized flour, PV – Peak viscosity, TV – Trough viscosity, FV – Final viscosity, BD_SqRt – Breakdown viscosity (squared-root-transformed), SB1 – Setback from trough viscosity, and SB2 – Setback from peak viscosity

Considering the data obtained across the two growing seasons, fiber (FI) and protein content (PR) appeared strongly and positively correlated with phenotypic and genetic correlations coefficients superior to 0.8 (Additional file 1: Table S1 and Additional file 2: Table S2). The high pairwise genetic correlation value (*r* > 0.8, Fig. 1) might indicate a common genetic basis for the phenotypic variation of these traits. Fiber content (FI) had the highest heritability value (*h*^*2*^ = 65%, across growing seasons) and setback from peak viscosity (SB2) the lowest (*h*^*2*^ = 31%, across growing seasons) (Table 1), indicative that there will be more difficulties in finding the genomic regions controlling SB2 than FI.

**Table 1:** Variance components and broad-sense heritability estimates for 11 quality traits measured in 132 maize inbred lines

Trait abbreviation^a^	Variance components^b^				*h*^*2*^ heritability ^c^(%)
	*σ* ^*2*^_p_	*σ* ^*2*^_g_	*σ* ^*2*^_g×y_	*σ* ^*2*^_error_	
PR	1.253	0.733	0.074	0.447	58
FI	0.045	0.029	0.001	0.014	65
FT	0.050	0.028	0.004	0.018	55
STL^d^	5.636	3.064	0.000	2.572	54
SIZEL^d^	615.987	238.055	46.222	331.710	39
PV	1.14E6	6.33E5	3.37E5	1.70E5	56
TV	2.74E5	1.19E5	3.77E4	1.18E5	43
FV	1.64E6	6.74E5	3.54E5	6.08E5	41
BD_SqRt^e^	167.218	103.016	36.195	28.007	62
SB1	9.53E5	3.29E5	1.58E5	4.67E5	34
SB2	8.22E5	2.51E5	1.89E5	3.82E5	31

*Quality traits measured in wholemeal maize flour from each of the 132 maize inbred lines evaluated in two growing seasons (2011 and 2012)*

^a^*Traits: PR* – *Protein, in %; FI* – *Fiber, in %; FT* – *Fat, in %; STL* – *Starch, in %; SIZEL* – *Mean particle size, in* μm*; PV* – *Peak (maximum) viscosity, in cP; TV* – *Trough (minimum) viscosity, in cP; FV* – *Final viscosity, in cP; BD_SqRt* – *Breakdown, in cP; SB1* – *Setback from trough viscosity, in cP; SB2* – *Setback from peak viscosity, in cP*

^b^ *Variance attributed to the individual terms of the statistical model: σ* ^*2*^_*p*_ *corresponds to the phenotypic variance; σ* ^*2*^_*g*_ *corresponds to the genotypic variance; σ* ^*2*^_*g×y*_ *corresponds to the interaction between inbred lines and growing seasons variance; σ* ^*2*^_*error*_ *(%) corresponds to the variance attributed to the block, row, column, and residual terms which altogether compose the error variance*

^c^ *h* ^*2*^ *=broad-sense heritability estimates obtained by fitting inbred lines as random terms in the statistical model across growing seasons*

^d^ *Traits values obtained from lyophilized flour*

^e^ *Breakdown values were squared-root transformed*

The analysis of the variance components was performed to access the proportion of variance attributed to differences among inbred lines and also to decide if the GWAS would be carried out using the adjusted means across years (in case the genotypic variance component was higher than the G × E interaction term) or on a year’s base. As shown in Table 1, the highest percentage of variance was typically due to differences between the inbred lines (*σ*^*2*^_g_), except for mean particle size (SIZEL), setback from trough viscosity (SB1), and setback from peak viscosity (SB2), where the error variance component was higher. The genotype-by-environment (G × E) interaction variance component (*σ*^*2*^_g × y_) was higher for traits related to maize flour pasting properties (viscosity parameters). Nevertheless, and for all traits analyzed, the variance component associated with differences between inbred lines was far greater than the variance component attributed to the effect of G × E interaction term (*σ*^*2*^_g_ / *σ*^*2*^_g × y_ > 1), and so GWAS was performed using the adjusted means across years. Additional information on the collection of maize inbred lines phenotypic values (range and mean ± standard deviation) for the quality-related traits evaluated in two growing seasons (2011 and 2012) can be found in Additional file 3: Table S3.

**Fig. 2:**
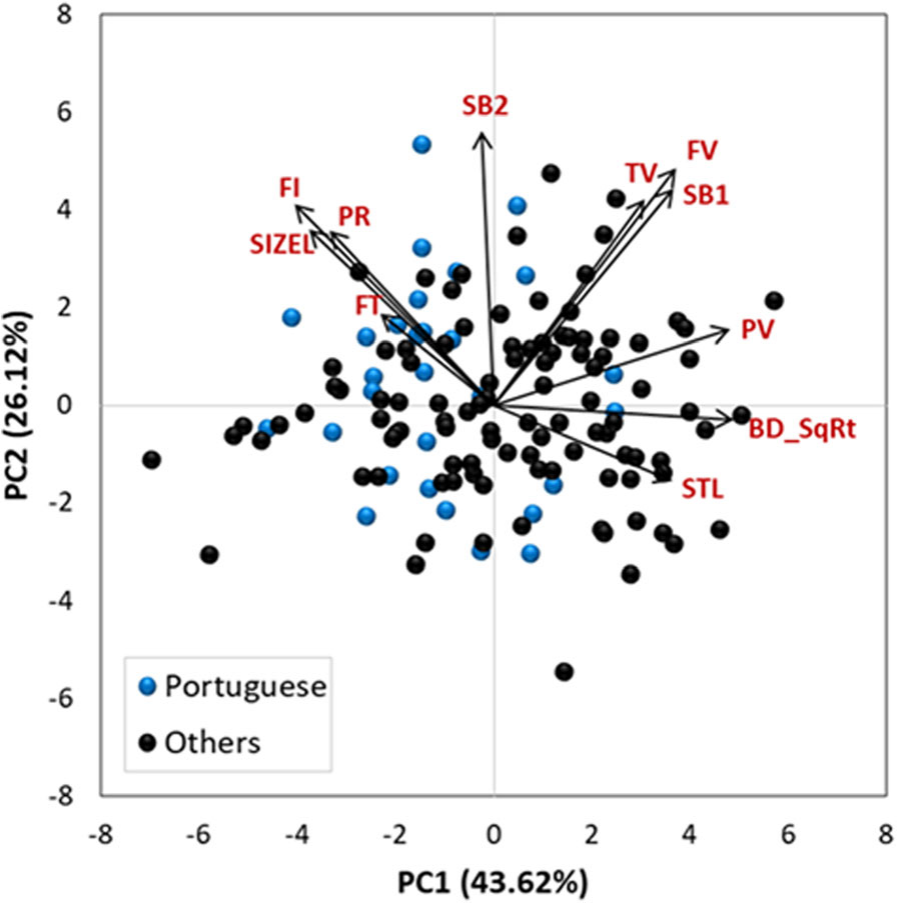
Principal component analysis (PCA) biplot for 11 quality traits measured in 132 maize inbred lines. PCA based on BLUP (best linear unbiased prediction) values across growing seasons. Blue circles correspond to inbred lines selected entirely from Portuguese landraces. Quality traits: PR –percentage of protein; FI – percentage of fiber; FT – percentage of fat; STL – percentage of starch in lyophilized flour; SIZEL – mean particle size in lyophilized flour; PV – peak (maximum) viscosity; TV – trough (minimum) viscosity; FV – final viscosity; BD_SqRt – squared-root transformed values of the breakdown viscosity; SB1 – setback from trough viscosity; SB2 – setback from peak viscosity

The maize inbred lines derived entirely from Portuguese traditional maize populations were characterized mainly by low breakdown and peak viscosity values, low starch content, and high protein, fiber, and mean particle size as can be seen in the biplot of the PCA (Fig. 2). In this analysis, the first two principal components (explaining a total of 69.74% of the variation) depicted a high diversity among the inbred lines of the association panel for the quality-related traits analyzed.

### Association panel genetic structure

**Fig. 3:**
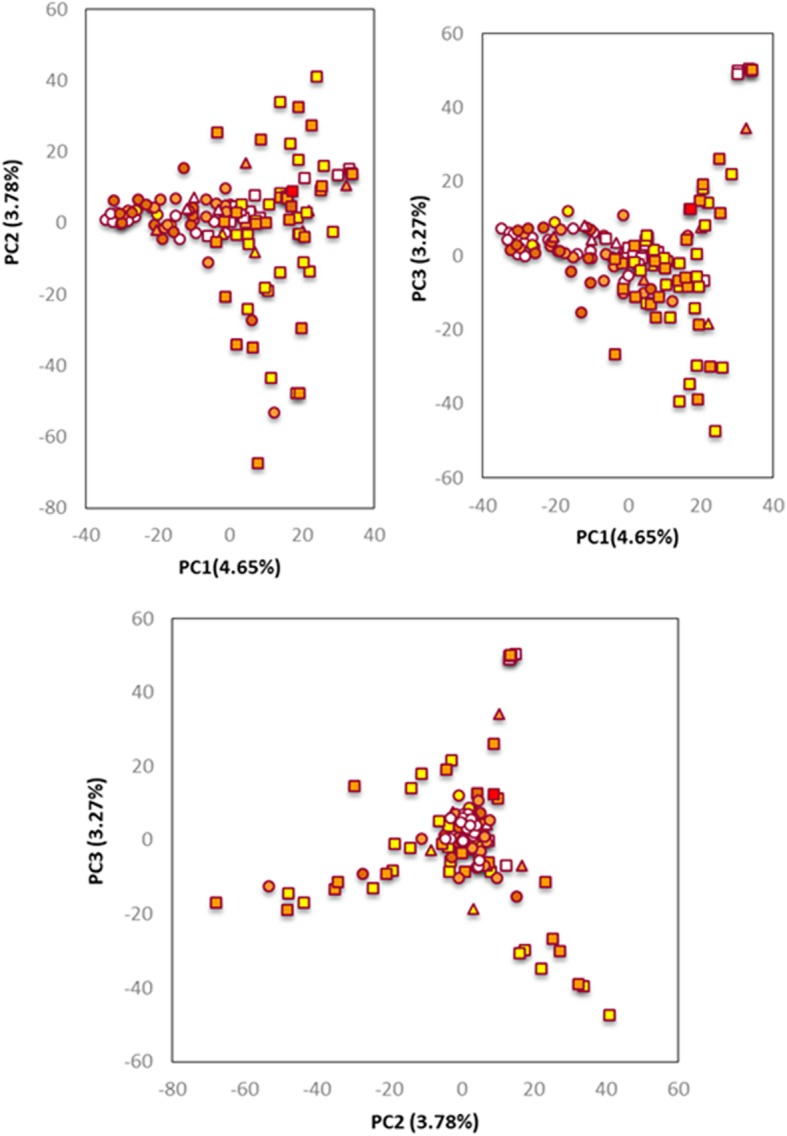
First three principal component scores plots from Eigenanalysis using 1821 SNPs in 132 maize inbred lines. Inbred lines coded by endosperm type: dent (squares), flint (circles), and intermediate (triangles); and kernel color (white, yellow, yellow-orange, orange, and red). The variance explained by each principal component is given in the axis heading

From the performed Eigenanalysis (Fig. 3) a wide dispersion of inbred lines was observed, with some separation according to kernel type (flint vs. dent) along the first principal component (PC1). The majority of the 29 lines selected directly from Portuguese traditional maize populations were clustered within the flint types.

The existence of strong genetic structure within the association panel can led to the identification of false positives in genome-wide association studies. In order to avoid spurious associations the GWAS was performed using a mixed linear model (MLM) and either kinship relationship (*K* matrix) or population structure (Eigenanalysis) was taken into account. The models that best corrects for population structure will have the inflation factor values closer to 1. After inspecting the observed inflation factors obtained for each tested model, the mixed linear model accounting for familial relatedness (*K* matrix) was selected as the best model (Additional file 4: Table S4). Therefore, the results reported below concern the results obtained using this model.

For all the studied major constituents of maize kernel (protein, fiber, fat, and starch content) and all the studied parameters affecting maize flour “breadability” (starch pasting properties and flour’s mean particle size), significantly associated single nucleotide polymorphism (SNP) markers were identified. In total, 72 unique SNPs were identified as being associated with at least one of the 11 quality-related traits analyzed across the two growing seasons (2011 and 2012) using a threshold –log_10_(*P*-value) = 4.

**Fig. 4:**
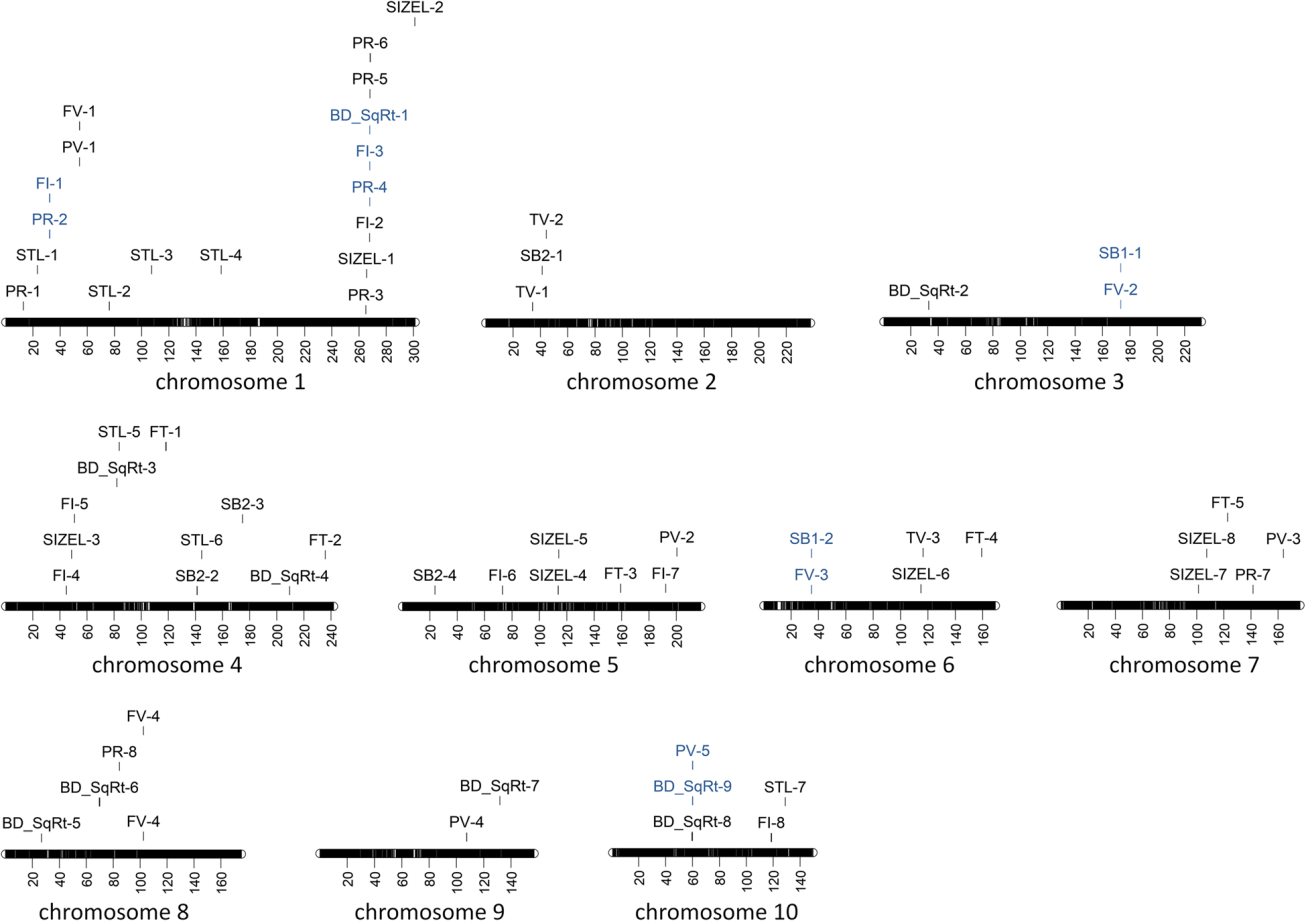
Chromosomal regions identified by genome-wide association for 11 quality traits measured in 132 maize inbred lines. Horizontal bars represent each of the 10 maize chromosomes. Genomic regions labelled accordingly to the trait, followed by a number identifying each individual region; vertical lines below correspond to the location of the genomic region associated with the trait variation in megabase pairs using the B73 reference genome (*Zea mays* B73 RefGen_v3). Co-localized regions associated with multiple traits are highlighted in blue. Quality traits: PR – percentage of protein; FI – percentage of fiber; FT – percentage of fat; STL – percentage of starch in lyophilized flour; SIZEL – mean particle size in lyophilized flour; PV – peak (maximum) viscosity; TV – trough (minimum) viscosity; FV – final viscosity; BD_SqRt – squared-root transformed values of the breakdown viscosity; SB1 – setback from trough viscosity; SB2 – setback from peak viscosity

The 72 SNPs corresponded to 57 genomic regions (Fig. 4). While some of the genomic regions were associated with several traits, the majority of the detected genomic regions were associated with a single trait. The number of regions identified for each quality trait varied from nine for breakdown viscosity (BD_SqRt) to two regions for setback from trough viscosity (SB1). Several regions controlling multiple traits were also identified (Fig. 4), which is consistent with the strong pairwise genotypic correlations observed between some of the traits, such as peak viscosity and breakdown viscosity, final viscosity, and setback from tough viscosity or protein and fiber content (Fig. 1).

The strongest associations were found for protein content and for several viscosity parameters (Additional file 5: Table S5). Specifically, the genomic region on chromosome 1 (32,314 kb to 32,548 kb) was strongly associated with protein content (PR), the genomic region on chromosome 5 (23,783,411 bp) was associated with setback from peak viscosity (SB2), and the genomic region on chromosome 10 (60,092 kb to 60,351 kb) was associated with peak viscosity (PV) and breakdown viscosity (BD_SqRt).

**Fig. 5:**
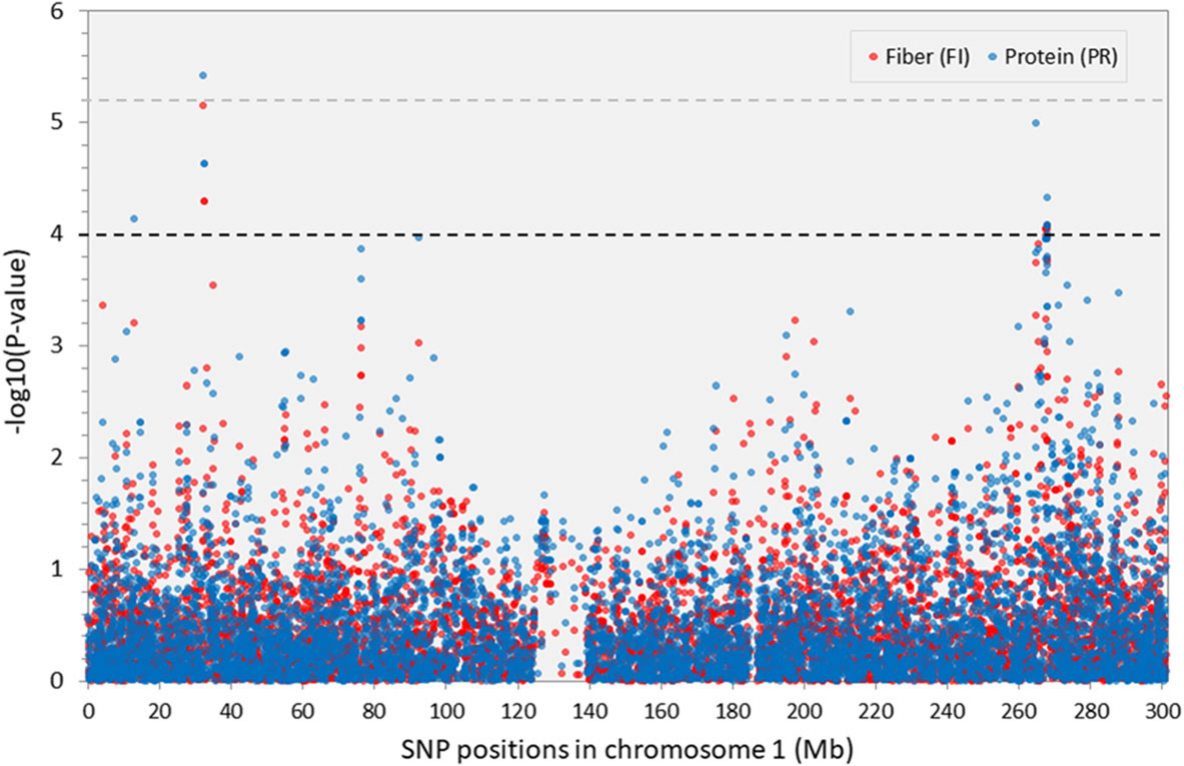
Manhattan plot showing GWAS results for wholemeal maize flour protein and fiber content on chromosome 1. *Y-axis* shows the −log_10_ *P*-values of each SNP, and *x*-axis shows their chromosomal positions. Horizontal black and grey lines represent the threshold of *P* = 1.00 × 10^− 4^, and the Bonferroni-corrected threshold of *P* = 6.45 × 10^− 6^, respectively

**Fig. 6:**
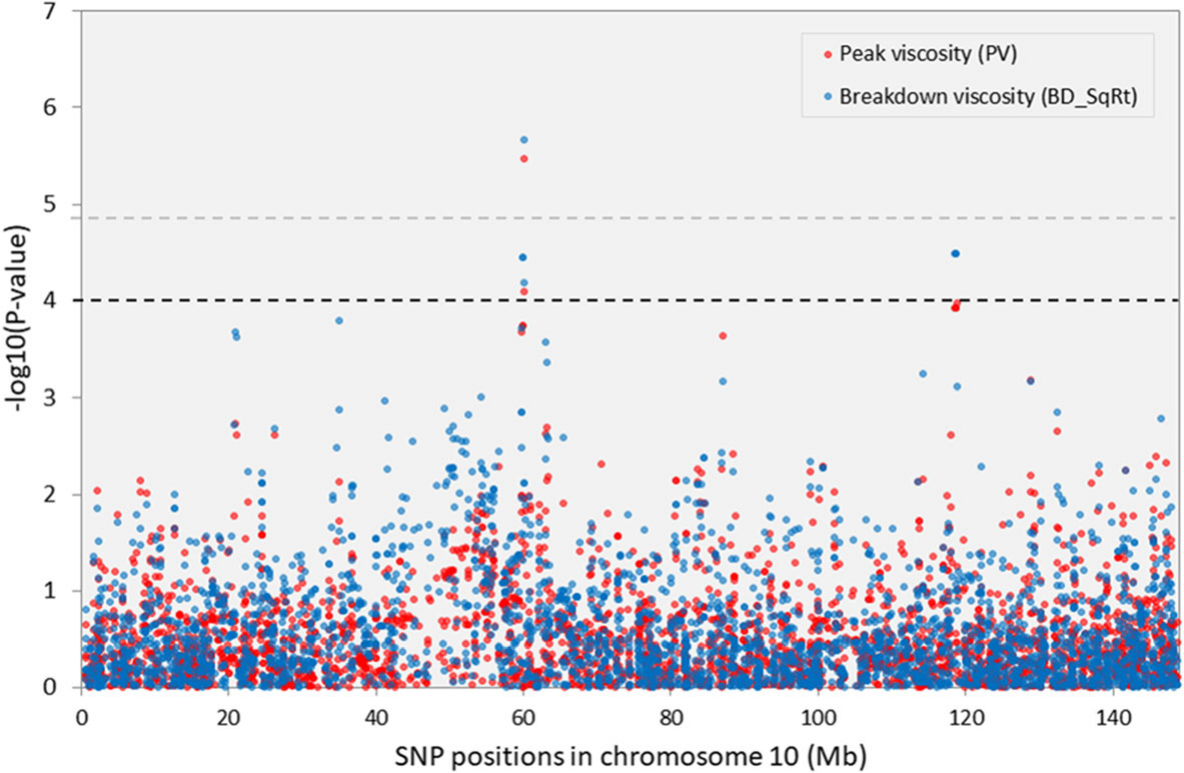
Manhattan plot showing GWAS results for wholemeal maize flour peak and breakdown viscosity on chromosome 10. *Y-axis* shows the −log_10_ *P*-values of each SNP, and *x*-axis shows their chromosomal positions. Horizontal black and grey lines represent the threshold of *P* = 1.00 × 10^− 4^, and the Bonferroni-corrected threshold of *P* = 1.44 × 10^− 5^, respectively

**Fig. 7:**
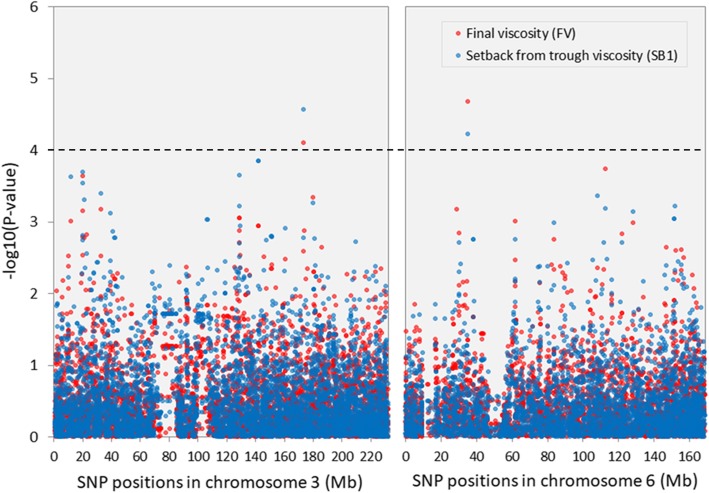
Manhattan plots showing GWAS results for setback from trough viscosity and final viscosity on chromosomes 3 and 6. *Y-axis* shows the −log_10_ *P*-values of each SNP, and *x*-axis shows their chromosomal positions. The horizontal black line represents the threshold of *P* = 1.00 × 10^− 4^

Five genomic regions were associated with multiple quality-related traits (Fig. 4); many of those traits were highly correlated (Fig. 1). Protein (PR) and fiber content (FI) were simultaneously associated in two different genomic regions on chromosome 1, one already described for PR, between 32,313 kilobase pairs (kb) and 32,548 kb and the other region between 267,849 kb and 267,886 kb (Fig. 5). The second region was also associated with breakdown viscosity (BD_SqRt) (Additional file 5: Table S5). Three other genomic regions were simultaneously associated with different traits related to flour’s pasting properties (viscosity profiles). Namely, one genomic region associated with breakdown viscosity (BD_SqRt) and peak viscosity (PV) on chromosome 10 (60,092 kb to 60,351 kb) (Fig. 6), and two other regions associated both with setback from trough viscosity (SB1) and final viscosity (FV) on chromosome 3 (173.419 kb to 173,420 kb) and chromosome 6 (34,978 kb to 35,091 kb) (Fig. 7).

### Proportion of variance explained and SNP effect size

For all the traits, each significant SNP-trait association only explained a small portion of the observed phenotypic variance. The portion of the observed phenotypic variance explained by each significant SNP-trait associations was bigger in the case of setback from peak viscosity (15.6%), and mean particle size (14.8%) (Additional file 6: Table S6).

By inspecting the significantly associated SNP effect sizes on the traits variation we were able to identify the most promising SNPs for marker-assisted-selection (SNPs with the strongest association with the traits and with higher effect on the trait variation). Namely, for setback from peak viscosity selection, the SNP (rs131504732) which can lead to a change in 18.89% in relation to the association panel mean value would be the most logical selection choice. For peak viscosity and breakdown viscosity, the SNP (rs131765763) that can lead to a change in 12.53 and 16.30%, respectively, in relation to the association panel mean value would be selected; and for protein, the choice would be the SNP (rs131232105) which can lead to a change in 4.53% in relation to the association panel mean value.

When considering the effect of the most frequent allele of the strongest SNPs associated with those quality traits and/or the SNPs that explained the biggest proportion of genetic variance on the maize inbred lines derived entirely from Portuguese populations, we observed that the frequency of the SNP variants in the Portuguese inbred lines was indeed directed toward an increase in protein, fiber, and mean particle size, and a decrease in starch, breakdown viscosity, and peak viscosity, as described before for the Portuguese derived lines. This can indicate a positive selection in the Portuguese maize germplasm toward the presence of the favorable alleles for protein content (SNP ID rs131232105), for fiber content (SNP ID rs132587158), and mean particle size (SNP ID rs131635762), and for alleles associated with a decrease in breakdown and peak viscosities values (SNP ID rs128531960) and decrease in starch content (SNP ID rs131186983). For example, in the strongest associated SNP on chromosome 1 for protein content (SNP ID rs131232105) that the unfavorable allele was only present in ~ 10% of the Portuguese lines.

### Identification of candidate genes

We further investigated the location of genomic regions for putative candidate genes using the B73 reference genome (*Zea mays* B73 RefGen_v3). The degree of linkage disequilibrium in the association panel determines the size of the genomic window to be considered when looking for candidate genes as it established the degree of mapping resolution. The average linkage disequilibrium (LD) decay for the genomic regions significantly associated with the quality-related traits was for our association panel 52.23 kb for LD r^2^ > 0.2. This value was highly variable across the genome extended to a maximum of 457 kb in a region of chromosome 10, spanning from 59,574 kb to 60,031 kb and identified as being associated with breakdown viscosity trait (BD_SqRt) (Additional file 5: Table S5). Using as reference the filtered gene set from the B73 RefGen_v3 assembly, a complete list of genes mapped within the significantly associated genomic regions identified in the GWAS for the 11 quality-related traits can be found in Additional file 7. A substantial proportion (66.67%) of the SNPs significantly associated with the quality-related traits was mapped within genes (48 out of 72 SNPs significantly associated with any trait; Additional file 7: Table S7). The degree of linkage disequilibrium around the genomic regions identified by GWAS allowed achieving a mapping resolution to the gene level in 40.35% of the cases (LD blocks where a single gene was identified, Additional file 7: Table S7).

In the frame of this work, it was not possible to describe all candidate genes located within the associated genomic regions in detail. We therefore restrict ourselves to describe those that were (1) located within regions where the strongest significant associations were detected or (2) located within regions associated with multiple quality-related traits.

The strongest SNP-trait associations detected, corresponding to three different genomic regions, were located on chromosomes 1, 5, and 10 (SNPs highlighted in Additional file 5: Table S5). They are the genomic region on chromosome 1 (32,314 kb to 32,548 kb) associated with protein content (PR) and fiber (FI), the genomic region on chromosome 5 (23,783,411 bp) associated with setback from peak viscosity (SB2), and the last on chromosome 10 (60,092 kb to 60,351 kb) associated with peak viscosity (PV) and breakdown viscosity (BD_SqRt). Some of these genomic regions harbored potential candidate genes for which we had no previous validation of their involvement with the quality-related traits analyzed. This was the case for the genomic regions on chromosome 1 strongly associated with PR and FI. Among the six protein-coding genes within this region, the SNP with the strongest association to both traits, rs131232105, was located within the GRMZM2G099528 gene. This gene codes for a putative endoplasmic reticulum transmembrane protein involved in the intracellular protein transport.

In the genomic region on chromosome 5, one SNP was strongly associated with setback from peak viscosity (SB2) (rs131504732, −log_10_ (*P*-value) = 5.846). This SNP was located within the GRMZM2G376743 gene, coding for a protein from the ARM repeat superfamily (PTHR33836:SF1). Finally, on chromosome 10, two SNPs were significantly associated with both peak viscosity (PV) and breakdown viscosity (BD_SqRt) (rs128531960 and rs131765763). Of those SNPs, the strongest SNP associated was rs131765763 (−log10 (P-value) = 5.468, for peak viscosity; and –log10 (P-value) = 5.671, for breakdown viscosity). The SNPs associated with those two traits were not mapped within any gene. Nevertheless, considering the LD decay around those SNPs, several genes were identified within the region: GRMZM2G079777, coding for a V-type proton ATPase subunit D protein, involved in the phagosome and in oxidative phosphorylation; GRMZM2G181192 (*glx1*), coding for glyoxylase1, involved in the pyruvate metabolism; and GRMZM2G079925 and GRMZM2G005938, both coding for pentatricopeptide repeat-containing proteins.

Furthermore, candidate genes were identified for the regions associated with multiple quality-related traits. On chromosome 1, besides the region described in the previous section strongly associated with protein content, another genomic region where several candidate genes were located was associated simultaneously with breakdown viscosity (BD_SqRt), fiber (FI), and protein (PR) content. This region spanned from 267,849 kb to 267,886 kb. Examples are the GRMZM2G127656 gene, which encodes a protein containing a zinc-finger domain of monoamine-oxidase A repressor R1 for fiber content, and the GRMZM2G022787 gene, which encodes for a pentatricopeptide repeat-containing protein for protein content.

Two regions were simultaneously associated with final viscosity (FV), and setback from trough viscosity (SB1), one on chromosome 3 spanning from 173,419 to 173,420 kb and the other on chromosome 6 spanning from 34,978 kb to 35,091 kb. In the first region (chromosome 3), the significant SNP (rs131180967, −log_10_ (*P*-value) = 4.571, for FV, and –log_10_ (P-value) = 4.103, for SB1) was mapped within the GRMZM2G452630 gene, coding for a serine hydroxyl-methyl-transferase related protein. In the second region (chromosome 6), the significant SNP (rs131176534, −log_10_ (P-value) = 4.675, for FV, and -log_10_ (P-value) = 4.224, for SB1) was mapped within the GRMZM2G045971 gene, coding for a preprotein translocase Sec, Sec61-beta subunit protein.

## Discussion

Breeding for maize improved quality is a complex task of great economic importance presently. The development of tools, such as molecular tools, to increase efficacy and efficiency of quality selection within breeding programs is becoming crucial. In the present study and by taking advantage for the first time of the underexplored Portuguese maize germplasm selected over centuries for food uses such as bread production, we conducted a genome-wide association study, identifying 57 genomic regions and candidate genes for 11 different maize kernel constituents and parameters affecting the maize flour “breadability”. These can now be used to support breeding programs for complex quality traits, difficult to select by conventional methods such as “breadability”.

Starch, proteins, and lipids are the three major food components in cereal-based food products, and interactions among them in a food system are of importance to functionality and quality [7, 8]. In a previous work we showed that Portuguese traditional maize populations considered to have high “breadability” were characterized among others by high levels of protein and fiber, and low breakdown viscosity values [19]. Additionally, the maize populations that produce better-quality bread have higher protein values and lower breakdown values when compared to commercial maize varieties [18]. Similarly, in the present work, the inbred lines derived from Portuguese maize populations were overall characterized by having a low breakdown and peak viscosity, and starch content; and a high fiber and protein content. Pasting properties of maize flour are considered important parameters in the preparation of different food products as they are related to starch swelling and gelatinization ability [20]. Besides starch, fiber and protein also influence the end-product quality. The higher protein content can potentially induce increased amounts of flour water absorption ratio and corresponding higher bread moisture. In fact, the crumb moisture has been identified [21] as a relevant attribute for consumer acceptability of maize-based bread. Fiber content can also have an impact on baked goods quality, contributing to dough viscosity, air entrapment and the improvement of loaf volume and texture [6].

The Portuguese inbred lines where mainly grouped with the lines with flint endosperm. This type of endosperm is usually associated to harder kernels. The endosperm or kernel hardness has been described as having a major impact on quality [21–23]. The size of the particles that are released from flour is directly related to the kernel hardness. Harder kernels or those richer in vitreous endosperm will yield larger particles than those that are softer [24]. With regard to the biochemical contribution to maize kernel hardness, both protein and starch composition are implicated, and specifically, the variation in zein classes has been linked to differences in hardness [23]. And both the content and composition of zeins are the key determinants to the protein quality and texture-related traits of the kernel [25].

### Putative pleiotropic regions associated with flour compositional and pasting properties

Pleiotropic effects (different traits affected by the same locus/loci) can hamper breeding efforts. In this work several regions controlling multiple traits were identified, which is consistent with the observation of strong pairwise phenotypic and genotypic correlations between some of the traits (such as peak viscosity and breakdown viscosity, final viscosity, and setback from tough viscosity or protein and fiber content). This detection of genomic regions associated with multiple traits variation could be due to pleiotropic effects. However, since several genes are mapped within some of those regions, as mentioned, for instance, in Karn et al. [26], fine mapping within these regions is still required in order to properly address whether a pleiotropic gene is responsible for both traits variation or whether the traits’ variation is due to two closely linked genes, and to investigate the possibility of independent selection among the correlated traits.

### Strong candidate genes for protein and fiber content in wholemeal maize flour

Although for the further development of selection tools is not necessary to identify the direct causal variant, being sufficient to identify the genomic region, the identification of strong candidate gene(s) with strong evidence of direct implication on a trait variation would reinforce the overall biological results. Several of the SNP-trait associations detected in the present study were located within or near an a priori candidate gene, which strengthened and validated the usefulness of the used association panel. For instance, in the current work the allelic variation for SNP ID rs128946933 (chromosome 1 at 267,974,184 bp; −log_10_ (*P*-value) = 4.002) was significantly associated with protein variation. This SNP is located within the GRMZM2G066749 gene. Recently, Chen et al. [27] demonstrated that this particular GRMZM2G066749 gene is the causative gene for *dek35* mutants. The mature *dek35* seeds have their protein content altered, exhibiting a significant decrease in seed dry weight and zein protein content [27]. Furthermore, the neighboring region to *dek35* was previously identified by Cook et al. [13] as being associated with the maize kernel protein content.

Also the SNP with the strongest association to both protein content and fiber content (Chr1: 32,313 kb to 32,548 kb; rs131232105) was located within the GRMZM2G099528 gene. This gene codes for a putative endoplasmic reticulum transmembrane protein involved in the intracellular protein transport. The intracellular transport of proteins into the lumen of the endoplasmic reticulum is crucial for the protein accumulation in the kernel. Maize kernel primary storage proteins are first sequestered and accumulated in the lumen of the endoplasmic reticulum, and are afterwards directly assembled into protein bodies [25]. This region is an example of a regions identified in this work harbored putative candidate genes for which we had no previous indication of their involvement with the quality-related traits analyzed. These “novel” regions with unforeseen candidate genes, not previously described as associated to the studied traits, might be due to the present use of a different association panel, harboring different genetic variability.

Several a priori candidate genes previously identified by others as associated with maize kernel compositional traits and starch pasting properties (e.g., [13–15]) were not detected in the present study. Examples are the *amylose extender1* (*ae1*) and s*hrunken2* (*sh2*), known also for their significant association with starch pasting properties [14]; *brittle endosperm2* (*bt2*); *shrunken1* (*sh1*); and *dull endosperm 1* (*du1*), known for a significant association with kernel compositional traits. The latter, *dull endosperm 1* gene (GRMZM2G141399, *du1*), encodes a starch synthase and is a determinant of the structure of endosperm starch in maize [28, 29]. This gene was found on chromosome 10 at a distance of approximately 46 kb and 564 kb downstream of two identified associated genomic regions with breakdown viscosity, one of the flour pasting properties studied in this work, and therefore not within the confidence intervals (59,574–60,031 kb and 60,092–60,351 kb, respectively). This observation may suggest that, at least for some regions, the rapid rate of LD decay observed in the present panel can partially explain the difficulty of detecting previously identified candidate genes named in other similar studies. Another example of a potential candidate gene for starch content, but not identified in the present study, is *brittle endosperm1* gene (GRMZM2G144081, *bt1*), coding a protein Brittle1 (Bt1) protein, involved in ADP-glucose transport into endosperm plastids and playing a role in starch biosynthesis [15]; or in the case of oil content in maize kernels, the acyl-CoA:diacylglycerol acyltransferase gene (GRMZM2G169089, *DGAT1–2*) [13, 30]. Indeed, the genotyping platform used on the current work screened several SNPs located within all the aforementioned candidate genes. Nevertheless, no association was detected between those SNPs and the maize kernel compositional traits or starch pasting properties on the present association panel. As pointed out by [13], several factors could be responsible for differences in position and quantity of quantitative trait loci (QTLs) detected between studies, including variation in allelic frequency, mapping resolution influenced by the magnitude of linkage disequilibrium in a population, marker density, environmental effects, and QTL analysis methods.

The rapid rate of LD decay observed in the present study in the SNPs associated with the quality-related traits evaluated suggests that a higher marker density would have been beneficial in the detection of other regions putatively linked to maize flour’s quality. Moreover, it is important to mention that the size of the collection of maize inbred lines used in this work most likely affected the power to detect significant marker-trait associations and the subsequent identification of genomic regions controlling the analyzed traits association. As reported in Yang et al. [31], using simulation studies, a collection of 155 diverse maize lines for association mapping was suitable for studying traits mainly controlled by major QTLs. Similarly, in the present work, the collection size should have been extended in order to increase the power to detect significant markers with moderate or even minor effects on the traits.

### Challenges and opportunities

This work reports the identification of 57 genomic regions associated with 11 different quality-related traits evaluated in wholemeal maize flour, highlighting candidate genes for the majority of those regions. However, novel regions, with no clear candidate genes, were also identified, which were not previously acknowledged using other germplasm collections studies.

Findings from GWAS provide valuable genetic information of trait architecture or candidate loci for subsequent validation [32]. Preliminary GWAS analysis should be complemented by statistical procedures to help prioritize GWAS results [33], such as pathway analysis of GWAS results to rank genes and pathways within a biological context. Additional follow-up analyses and experiments may even be required to pinpoint the causal genes [34]. In the present study, the SNPs strongly associated with the traits analyzed and/or the SNPs from which the allelic variant was found to contribute to the larger phenotypic effects should be prioritized as candidate genomic regions for marker development to support selection activities, especially for the quality-related traits difficult to measure/assess. The final objective is to develop the necessary expedited tools to implement routine quality selection (such as for “breadability”) into maize breeding programs. As an example, by using marker-assisted selection, a few nutritional trait-associated genes or QTLs (for maize protein quality, oil content and provitamin A levels) have been recently introgressed into elite maize lines for their quality improvement [5].

Nevertheless, prior to that, the significant marker-trait associations detected in the current work need to be validated. Future work will concentrate on the validation of the results retrieved in this work by sequencing those regions on contrasting maize populations for the given trait. Since the actual materials used for the manufacturing of maize-based bread are the traditional maize populations, these are the ideal independent materials to proceed with the sequencing validation.

## Conclusions

In this work, a genome-wide association approach was carried out to identify genomic regions controlling the variation of maize kernel major constituents (protein, fiber, fat, and starch content) and parameters affecting the maize flour “breadability” (starch pasting properties and flour’s mean particle size). For that, a unique maize inbred collection containing lines partially derived from Portuguese maize populations was used given that Portuguese traditional maize populations have been developed through centuries adapted both to the local environment and food uses, in particular, for *broa* production.

The inbred lines derived from Portuguese maize populations were overall characterized by having a low breakdown and peak viscosity, and starch content; and a high fiber and protein content. A putative positive selection toward the presence of the favorable alleles for protein content, for fiber content, and mean particle size, and for alleles associated with a decrease in breakdown and peak viscosities values and a decrease in starch content was observed in the inbred lines derived from Portuguese traditional maize populations.

This work allowed for identifying relevant regions on the maize genome affecting maize kernel composition and flour pasting behavior, and identifying candidate genes for the majority of the flagged genomic regions. A total of 57 genomic regions were detected and candidate genes underlying the majority of those regions were identified. Moreover it was observed that the majority of the detected genomic regions were associated with a single trait. The traits for which the strongest and stable associations were found were protein, breakdown viscosity and peak viscosity. For those the strongest SNP-trait association could result in changes from more than 4% in protein content to more than 10% in the case of the viscosity parameters. Importantly, this work allowed reducing the gap towards the development of selection tools to support breeding for these complex quality traits, such as “breadability”.

## Methods

### Plant material selection

In this work, we took advantage of the diverse inbred lines developed in the past by the extinct NUMI (Núcleo de Melhoramento de Milho) combining Portuguese germplasm with foreign (mainly US) germplasm. These inbred lines (developed by single seed descent) are currently conserved at the National Portuguese Plant Germplasm Bank (Banco Português de Germoplasma Vegetal - BPGV, Braga, Portugal). The maize inbred line collection used in this study was assembled observing a significant representation of lines selected from Portuguese traditional maize populations (29 lines) and lines with a mixed Portuguese × foreign origin (Additional file 8: Table S8).

From a total of 164 different maize inbred lines sowed on the field trials, only 132 yielded sufficient kernels to proceed with their quality analysis. Additional details on their recorded pedigree can be found in Additional file 8: Table S8. Thirty-six of the yielding lines have white kernel, further divided into 20 with flint endosperm, three intermediate and 13 with dent endosperm. The remaining 96 inbred lines have a kernel color ranging from yellow to red, further divided into 37 with flint endosperm, eight intermediate, and 51 with dent endosperm (Additional file 9: Table S9).

### Field characterization and experimental design

The inbred lines were evaluated at the Coimbra site (40°13′0.22″N, 8°26′47.69″W) in Portugal during the 2011 and 2012 growing seasons, using an organic agriculture converted field. The conversion started in 2011 and the field was considered to be fully managed under an organic agriculture system by 2012. This site is part of the Mondego River irrigation perimeter, a very high-yielding maize area where the average maize hybrids yield is 14.5 Mg.ha^− 1^ [35]. It is located 50 km from the seacoast, with an altitude of 25 m. Its alluvial soils are characterized at 0–20 cm and 20–40 cm, respectively, by a pH of 5.65 and 5.75; a percentage of soil with a particle size less than 0.2 mm diameter of 83.37 and 82.84%; and an organic matter percentage of 2.91 and 2.55%. Agricultural practices were similar in both growing seasons, but sowing and harvest dates differed between growing seasons. Sowing took place on April 28 and May 11 and the harvests took place on September 28 and November 6 in 2011 and 2012, respectively.

In each year, the maize inbred lines were evaluated using a randomized complete block design, with two blocks (replicates). Information on the spatial distribution of the plots was also recorded (field row and columns coordinates). Each plot consisted of two rows 7.2 m long (6.4 m planted row plus 0.8 m border space between two planted rows), with an inter-row distance of 0.75 m. Each plot was overplanted by hand and thinned at the V7 growth developmental stage to achieve a plant density of approximately 50,000 plants ha^− 1^. Plots were both mechanically weeded and hand-weeded when needed and managed following common agricultural practices for maize in the region. Pollination was controlled within each plot. All the plots were harvested by hand. After harvest, ears were dried at 30–35 °C in an oven (Memmert Model UFE 800, Memmert GmbH + Co. KG, Germany) until a ~ 15% in moisture was reached. The ears were then shelled, and the kernel collected per plot basis was packed in paper bags and kept at 4 °C until further analysis.

### Sample preparation and phenotypic data acquisition

A seed sample from each of the harvested plots was used for quality determinations in the two years/growing seasons. Eleven nutritional-related and technological-related traits were measured in wholemeal maize flour. Wholemeal maize flour was obtained from all the seed samples using a Falling number 3100 mill (Perten Inc., Sweden) with a 0.8 mm screen. In order to prevent/minimize the enzymatic action and subsequent alteration of the flour properties, flour samples were also lyophilized using Cientificolab® equipment built for pilot-scale lyophilization of food commodities. For that, each sample was individually placed in a flask (height 3.7 cm, diameter 4.2 cm) and then freeze-dried for long-term preservation.

#### Nutritional-related traits

Flour protein (PR), fiber (FI), and fat (FT) content were determined for each non-lyophilized sample by near-infrared reflectance (NIR) spectroscopy (Percon Inframatic 8620, Perten Inc., Sweden), with calibrations for non-lyophilized samples supplied by the manufacturer. Values for protein, fiber, and fat corresponded to the mean value of up to two technical replicates. The total starch content was determined in lyophilized (STL) (both 2011 and 2012 growing seasons) and non-lyophilized (ST) (only the 2012 growing season) samples using Fourier Transform Near-Infrared Reflectance (FT-NIR) spectroscopy (FT-NIR MPA, Bruker Optics, Germany), with calibrations for non-lyophilized samples supplied by the manufacturer. Values for total starch content obtained from the 2012 growing season lyophilized and non-lyophilized samples were further used to test whether both datasets were correlated (phenotypic correlation between datasets). Values for total starch content (non-lyophilized (ST) and lyophilized (STL) samples) corresponded to the mean value of two to four technical replicates. Protein, fiber, fat, and starch content was expressed as a percentage (%).

#### Technological-related traits

The maize flour Particle Size Index (PSI) was also determined using FT-NIR spectroscopy (FT-NIR MPA, Bruker Optics, Germany). For the 2011 growing season, only the mean for particle size in lyophilized samples (SIZEL) was measured. For the 2012 growing season, both mean particle size in non-lyophilized (SIZE) and lyophilized flours (SIZEL) were determined. Values for mean particle size (non-lyophilized (SIZE) and lyophilized (SIZEL) samples) corresponded to the mean value of two to four technical replicates. The calibration models for PSI FT-NIR analysis were obtained using the particle size values measured in a subset of 30 non-lyophilized samples according to the AACC method 55–40.01:1999 [36], with a Malvern multi-channel laser light-scatter instrument (Malvern Instruments Ltd., England). Values for mean particle size obtained from lyophilized and non-lyophilized samples from the 2012 growing season were further used to test whether both datasets were correlated (phenotypic correlation between datasets). After calibration, the mean particle size volume value, or D [3, 4], retrieved from the particle size distribution, was used as an average measure of the particle size of each sample and was expressed in μmeters.

Maize flour pasting properties were evaluated by recording their viscosity profiles using a Rapid Visco Analyser (RVA) (Newport Scientific, Australia). The viscosity profiles were obtained on non-lyophilized samples according to [37] at 15% solids, using the following heating and cooling cycle set: (1) holding at 50 °C for 2 min, (2) heating to 95 °C for 4.5 min, (3) holding at 95 °C for 4.5 min, (4) cooling to 50 °C for 4 min, (5) holding at 50 °C for 10 min. The RVA paddle speed was set at 960 rpm for the first 10 s of the test, after which the speed was changed to 160 rpm. The following traits were recorded: Peak (or maximum) (PV), trough (or minimum) (TV), and final (FV) viscosities. The breakdown (BD) was calculated as peak viscosity-trough viscosity, setback from trough viscosity (SB1) as final viscosity - trough viscosity, and setback from peak viscosity (SB2) as final viscosity - peak viscosity. Up to two technical replicates of the viscosity profiles were taken for each sample. All the viscosity and viscosity-related traits were expressed in cPoise units.

### Phenotypic data analysis

#### Phenotypic data quality control

Quality control was performed for each year/growing season, on the data collected per genotype individually from each of the two replicated plots. Graphical inspection of residuals was used to assess normality (Q-Q plot), homogeneity of variance (residuals versus fitted values), and identify outliers. Observations were flagged for closer inspection when they exceeded 1.5 times the interquartile range, and when the standardized residuals after mixed model analysis were extreme. One of the traits (breakdown viscosity, BD) required a squared-root-transformation to stabilize the variance. All analyses were done using Breeding View software [38], available through the IBP Breeding Management System (IBP Breeding Management System Version 3.0.9, 2015).

#### Single/individual growing season analysis

A phenotypic analysis was performed per single environment trial: 1) the estimates of genetic variances (and covariances between traits) and of heritability were obtained according to Oakey et al. [39], and 2) adjusted trait means for all the inbred lines were calculated.

In detail, single trait-single growing season analysis, using mixed models, was performed using the “Single trait field trial analysis” pipeline of Breeding View, selecting the model for resolvable row-column design as implemented in the software. The statistical model includes an intercept, a fixed block effect, a random row and column effects (nested within blocks), a genotypic effect (fixed or random; see the explanation that follows), and a residual. The Field trial analysis node in Breeding View performs two mixed model analyses: In the first step (Step 1) the inbred lines (genotypes) were fitted as a random term, while in the second step (Step 2) the inbred lines were fitted as a fixed term. The Step 1 model is used to obtain estimates of variance parameters. From Step 1 the heritability, as well as the best linear unbiased predictors (BLUPs), was calculated for each inbred line (and correlations between BLUPs of different traits used to obtain estimates of genetic correlations between traits). In Step 2, structural variance components (rows and column variances) are fixed to those estimated in Step 1, and by including the inbred lines as a fixed term, the best linear unbiased estimators (BLUEs) for each inbred line were produced.

#### Multi-environment/growing seasons trial analysis

For each quality trait, a multi-environment trial analysis was also performed to assess the consistency across growing seasons. The analysis of variance was carried out using the residual maximum likelihood (REML) variance components analysis procedure in Genstat software (Genstat® for Windows, 18th edition, [40]). The mixed model included growing seasons (fixed), maize inbred lines, and season by line interaction (fixed or random) while blocks, rows, and columns were treated as random terms and nested within growing seasons. Similar to what was already described for the single trial analysis, in the multi-environment trial analysis, BLUPs and BLUEs were calculated for each inbred line across growing seasons. BLUPs were used on principal component analysis (PCA) to assess genetic correlations between traits and BLUEs were used as input phenotypic data in the association mapping analysis, for the combined analysis across growing seasons.

### Genotypic data acquisition

DNA was isolated from adult leaves from each maize inbred line using a modified CTAB procedure described in [41]. DNA quality was accessed using a 0.8% SeaKem® LE Agarose gel (Cambrex Bio Science Rockland, Inc., USA) stained with SYBR® Safe (Invitrogen, USA). DNA quantification was done using a spectrophotometer Nanodrop ND-2000C (Thermo Scientific, USA). An additional step for polysaccharides removal [42] was added when the ratio 260/230 nm wavelength was inferior to 1.6 to avoid the interference of these contaminants on Single Nucleotide Polymorphism (SNP) genotyping. DNA concentration for all inbred lines was set to 50 ng/μl and genotyped with the Illumina MaizeSNP50 BeadChip array [43]. The genotyping array procedure and alleles scoring was conducted by the genotypic service provider (TraitGenetics GmbH, Gatersleben, Germany). This array allowed the screening of 17,520 genes (since 33,417 of the SNPs present in this array are located on 17,520 genes and 16,168 SNPs are located in intergenic regions) [43].

The position of each marker along the maize B73 reference genome was updated from the markers’ coordinates available when the MaizeSNP50 BeadChip was originally designed (B73 reference genome version 1) to the coordinates in the released B73 reference genome version 3. These coordinates were taken from the maize genome browser, via the MaizeGDB database ([44], www.maizegdb.org).

### Genotypic data analysis

#### Genotypic data quality control

Genotypic data quality control was performed by removing SNP markers and inbred lines with more than 25% of missing data. SNPs called as heterozygous were set as missing data (0.93% of the total SNP calls). Moreover, markers with a minor allele frequency (MAF) smaller than 5% were removed. After this filter, a total of 48,772 SNPs remained and were used for the association mapping analysis.

#### Genetic structure analysis

A subset of 1821 SNPs, evenly distributed across the genome (corresponding approximately to 1 SNP per Megabase pairs, Mb), was used to calculate principal components to study the population structure among inbred lines and to calculate the kinship matrix to estimate pairwise genetic relatedness among inbred lines as implemented in Genstat software (Genstat® for Windows, 18th edition, [40]).

### Association mapping analysis

Given that for all the quality-related traits under study, the variance components for genotype-by-environment (G × E) interaction (σ^2^_g × y_) were much smaller than the genotype variance component (σ^2^_g_), univariate association analysis was carried out using the adjusted means for field trial design (BLUEs) obtained across growing seasons. Genome-wide association studies were conducted with the Genstat software using the available genotypic (SNPs scored with the MaizeSNP50 BeadChip array) and quality data (11 quality-related traits) measured in 132 maize inbred lines. The Genstat software performs association mapping in the mixed model framework, fitting markers as fixed and inbred lines as random terms using REML [45].

Three different models were tested to detect significant marker-trait associations: the naïve model [*Phenotype* = *SNP* + (*Genotype* + *Error*)], that neither accounts for population structure nor familial relatedness; a model accounting for population structure (*Q*) using 15 principal components from PCA [*Phenotype* = *Q* + *SNP* + (*Genotype* + *Error*)]; and a model accounting for familiar relatedness (*K*) [*Phenotype* = *SNP* + *Genotype* + *Error*] with Genotype random effects structured following a kinship matrix *K*. For each chromosome, a different kinship matrix was calculated where only the SNPs located on the other nine maize chromosomes were used to calculate the kinship matrix [46, 47].

The inspection of the inflation values for each model and the quantile-quantile (Q-Q) plots of the respective *P*-values allowed to informally assess to what extent the models accounted for genetic structure/relatedness among the inbred lines and therefore guarded against false discoveries. Models resulting in an inflation factor near 1 perform better, and quantile-quantile (Q-Q) plots should show few *P*-values that deviate from the expected uniform distribution that holds under the null hypothesis (i.e., no association). The observed *P*-values (on a –log_10_ scale) of each SNP were plotted against their chromosomal positions so they produce Manhattan plots. A threshold of –log_10_ (*P*-value) = 4 was set to identify significant marker-trait associations. Given that the association panel used was not that large and looking at the background noise in the obtained Manhattan plots, the threshold of -log10 (P-value) = 4 seemed reasonable not to avoid losing potentially interesting regions, at the expense of applying a more conservative type of correction such as Bonferroni. Examples of other maize studies with similar association panel sizes and number of markers using the same threshold are the works of Liu et al. [48, 49]. The effect of the minor frequency SNP variant, reported in relation to the most frequent allele reference, was calculated.

### Post-GWAS procedures

#### Local linkage disequilibrium and candidate genes identification

A local linkage disequilibrium (LD) study was performed to define chromosomal regions to search for candidate genes for the traits under analysis.

This procedure was done in two steps: In Step 1, the average intra-chromosomal LD was estimated as the squared correlation coefficient r^2^, after correcting for population structure using the principal component scores from Eigenanalysis, as implemented in Genstat software. For this calculation, the same subset of 1821 SNPs previously used for the genetic structure analysis was employed. LD decay was visualized per chromosome by plotting r^2^ against the physical mapping distance in Mb. A threshold for LD decay (r^2^ = 0.1) was used to estimate the average genetic distance for which markers were considered to be no longer correlated. In Step 2, a genomic window around each SNP location significantly associated with the traits analyzed was established by subtracting and adding the average genetic distance for LD decay (r^2^ > 0.1), estimated in Step 1. All the SNP markers located within those windows were then used to estimate the local LD decay. At this point, a stricter threshold of r^2^ = 0.2 was considered. The markers’ positions flanking each local LD block were further used as query positions on the maize genome browser, via MaizeGDB (https://www.maizegdb.org/gbrowse/), to retrieve the list of candidate genes mapped within those genomic regions.

The genome sequence of the maize inbred line B73 (*Zea mays* B73 RefGen_v3) was used as the reference genome for candidate gene analyses [50]. The functional annotation of the genes under the identified genomic regions was retrieved via Phytozome ([51], Phytozome 11, version AGPv3 - *Zea mays* Ensembl-18) using the gene model identifier as the query. KEGG: Kyoto Encyclopedia of Genes and Genomes database [52] was used to retrieve information on the pathways where the candidate genes could be involved.

## Additional files

Additional file 1: **Table S1.** Pearson correlation coefficients among quality traits measured in wholemeal flour of 132 maize inbred lines. In Table S1 one can find the pairwise Pearson correlations coefficients between the 11 quality trait evaluated and the *P*-value of the 2-tailed test for each growing season. (DOCX 28 kb)

Additional file 2: **Table S2.** Estimated genetic correlations among quality traits measured in wholemeal flour of a collection of 132 maize inbred lines. In Table S2 one can find the estimated pairwise genetic correlation between the 11 quality trait evaluated for each growing season. (DOCX 22 kb)

Additional file 3: **Table S3.** Phenotypic values (range, and mean ± standard deviation) for 11 quality traits measured in 132 maize inbred lines. In Table S3 one can find for the 11 quality trait evaluated the summary statistics on phenotypic data for each growing season and across growing seasons. (DOCX 24 kb)

Additional file 4: **Table S4.** Observed inflation factors for the models tested in genome-wide association (GWAS) analysis. In Table S4 one can find the inflation factors for each of the three different models that were tested to detect significant marker-trait associations: the naïve model, a model accounting for population structure; and a model accounting for familiar relatedness. (DOCX 21 kb)

Additional file 5: **Table S5.** Significant SNP-trait associations from a genome-wide association study for 11 quality traits in wholemeal maize flour. In Table S5 one can find the genome-wide association study results for the 11 quality trait evaluated based on the information combining the phenotypic data obtained in two growing seasons with the genotypic data obtained from the Illumina MaizeSNP50 BeadChip array using 132 maize inbred lines. Additionally, Table S5 also encloses information on the genomic regions associated to quality-related traits previously identified by other authors. (XLSX 22 kb)

Additional file 6: **Table S6.** Percentage of associated SNPs with opposite effects on each traits value, and the range of phenotypic variance explained. In Additional file 6: Table S6 one can find the summarized information on the overall abundance of alleles increasing and decreasing the trait value in the inbred line collection; information on the range of phenotypic variance explained by significantly associated SNPs; information on the effect of rare allele explaining the largest phenotypic variance. (DOCX 23 kb)

Additional file 7: **Table S7.** Candidate genes underlying the genomic regions associated with 11 quality traits in wholemeal maize flour. In Additional file 7: Table S7 one can find the information on the candidate gene annotations and information on its function. (XLSX 29 kb)

Additional file 8: **Table S8.** Maize inbred lines with available quality data, known pedigree, kernel color, and endosperm type. In Additional file 8: Table S8 one can find the list of 132 inbred lines that yielded sufficient kernels to proceed for quality analysis. The underlined inbred lines correspond to the lines derived entirely from Portuguese traditional maize populations, according to the Portuguese Plant Germplasm Bank records. (DOCX 34 kb)

Additional file 9: **Table S9.** Number of maize inbred lines grouped accordingly to their kernel color and endosperm type. In Additional file 9: Table S9 one can find the summary of the number of inbred lines with the different kernel colors and endosperm types. (DOCX 21 kb)

## Abbreviations

BD: Breakdown viscosity
BD_SqRt: Breakdown viscosity, squared-root-transformed values
BLUEs: Best linear unbiased estimators
BLUPs: Best linear unbiased predictors
FI: Fiber content
FT: Fat content
FT-NIR: Fourier Transform Near-Infrared Reflectance
FV: Final viscosity
G × E: Genotype-by-environment
GWAS: Genome-wide association study
LD: Linkage disequilibrium
MAF: Minor allele frequency
PCA: Principal component analysis
PR: Protein content
PSI: Particle Size Index
PV: Peak (or maximum viscosity)
Q-Q: Quantile-quantile
QTL(s): Quantitative trait locus (loci)
REML: Residual maximum likelihood
RVA: Rapid Visco Analyser
SB1: Setback from trough viscosity
SB2: Setback from peak viscosity
SIZE: Mean particle size
SIZEL: Mean particle size in lyophilized samples
SNP(s): Single nucleotide polymorphism(s)
ST: Starch content
STL: Starch content in lyophilized samples
TV: Trough (or minimum) viscosity
