# Supplementary material for: Genome-wide association study for kernel composition and flour pasting behavior in wholemeal maize flour
Source: BMC Plant Biol. 2019 Apr 2;19:123. doi: 10.1186/s12870-019-1729-7 (PMC6444869; doi:10.1186/s12870-019-1729-7)
Supplement: Supplementary file 1 — Table S1. Pearson correlation coefficients among quality traits measured in wholemeal flour of 132 maize inbred lines. In Table S1 one can find the pairwise Pearson correlations coefficients between the 11 quality trait evaluated and the P-value of the 2-tailed test for each growing season. (DOCX 28 kb) [file 12870_2019_1729_MOESM1_ESM.docx]

*Additional file 1*

**Table S1. Pearson correlation coefficients among quality traits measured in wholemeal flour of 132 maize inbred lines.**

|  |  | PR | FI | FT | ST | STL | SIZE | SIZEL | PV | TV | FV | BD_SqRt | SB1 | SB2 |
| --- | --- | --- | --- | --- | --- | --- | --- | --- | --- | --- | --- | --- | --- | --- |
| PR | r | - | **0.817** | -0.168 | NA | -0.512 | NA | 0.319 | -0.302 | 0.023 | -0.039 | -0.429 | -0.114 | 0.294 |
|  | P-value | - | ******* | ns | NA | *** | NA | ** | ** | ns | ns | *** | ns | ** |
| FI | r | **0.866** | - | 0.332 | NA | -0.624 | NA | 0.650 | -0.373 | 0.096 | -0.040 | -0.606 | -0.1662 | 0.388 |
|  | P-value | ******* | - | *** | NA | *** | NA | *** | *** | ns | ns | *** | ns | *** |
| FT | r | -0.066 | 0.394 | - | NA | -0.320 | NA | 0.519 | -0.149 | 0.035 | -0.038 | -0.272 | -0.091 | 0.124 |
|  | P-value | ns | *** | - | NA | ** | NA | *** | ns | ns | ns | ** | ns | ns |
| ST | r | -0.456 | -0.553 | -0.333 | - | NA | NA | NA | NA | NA | NA | NA | NA | NA |
|  | P-value | *** | *** | ** | - | NA | NA | NA | NA | NA | NA | NA | NA | NA |
| STL | r | -0.313 | -0.443 | -0.352 | **0.807** | - | NA | -0.410 | 0.314 | 0.025 | 0.224 | 0.449 | 0.353 | -0.025 |
|  | P-value | ** | *** | ** | ******* | - | NA | *** | ** | ns | * | *** | *** | ns |
| SIZE | r | 0.461 | 0.681 | 0.471 | -0.324 | -0.319 | - | NA | NA | NA | NA | NA | NA | NA |
|  | P-value | *** | *** | *** | ** | ** | - | NA | NA | NA | NA | NA | NA | NA |
| SIZEL | r | 0.590 | **0.804** | 0.557 | -0.526 | -0.339 | **0.765** | - | -0.288 | 0.197 | -0.097 | -0.602 | -0.305 | 0.197 |
|  | P-value | *** | ******* | *** | *** | ** | ******* | - | *** | ns | ns | *** | ** | ns |
| PV | r | -0.374 | -0.517 | -0.326 | 0.419 | 0.447 | -0.362 | -0.416 | - | **0.770** | **0.766** | **0.850** | 0.677 | -0.006 |
|  | P-value | ** | *** | ** | *** | *** | ** | *** | - | ******* | ******* | ******* | *** | ns |
| TV | r | -0.074 | -0.151 | -0.168 | 0.111 | 0.289 | 0.007 | 0.012 | **0.758** | - | **0.758** | 0.367 | 0.535 | 0.312 |
|  | P-value | ns | ns | ns | ns | * | ns | ns | ******* | - | ******* | *** | *** | ** |
| FV | r | -0.073 | -0.167 | -0.186 | 0.271 | 0.342 | -0.048 | -0.129 | **0.780** | **0.814** | - | 0.553 | **0.894** | 0.555 |
|  | P-value | ns | ns | ns | * | ** | ns | ns | ******* | ******* | - | *** | ******* | *** |
| BD_SqRt | r | -0.484 | -0.672 | -0.416 | 0.477 | 0.453 | -0.538 | -0.611 | **0.928** | 0.529 | 0.659 | - | 0.610 | -0.200 |
|  | P-value | *** | *** | *** | *** | *** | *** | *** | ******* | *** | *** | - | *** | ns |
| SB1 | r | -0.063 | -0.154 | -0.174 | 0.316 | 0.324 | -0.070 | -0.185 | 0.712 | 0.613 | **0.958** | 0.636 | - | 0.630 |
|  | P-value | ns | ns | ns | ** | ** | ns | ns | *** | *** | ******* | *** | - | *** |
| SB2 | r | 0.398 | 0.451 | 0.155 | -0.142 | -0.062 | 0.445 | 0.374 | -0.113 | 0.250 | 0.503 | -0.232 | 0.562 | - |
|  | P-value | *** | *** | ns | ns | ns | *** | ** | ns | * | *** | ns | *** | - |

*The phenotypic correlations were calculated independently for each growing season evaluated (year 2011 and year 2012). The values above the diagonal correspond to the phenotypic correlations among quality traits measured in the first growing season (2011); values below the diagonal correspond to the phenotypic correlations among quality traits measured in the second growing season (2012). In bold are highlighted the strong phenotypic correlations (|r| > 0.8).*

*r corresponds to the Person correlation coefficient; P-value corresponds to the significance level of correlations indicated as: ns - non-significant; * - significant at P < 0.05; ** - significant at P < 0.01; *** - significant at P < 0.001.*

*NA corresponds to data not available.*

*Quality traits: PR – percentage of protein; FI – percentage of fiber; FT – percentage of fat; ST – percentage of starch in non-lyophilized flour; STL – percentage of starch in lyophilized flour; SIZE – mean particle size in non-lyophilized flour; SIZEL – mean particle size in lyophilized flour; PV – peak (maximum) viscosity; TV – trough (minimum) viscosity; FV – final viscosity; BD_SqRt – squared-root transformed values of the breakdown viscosity; SB1 – setback from trough viscosity; SB2 – setback from peak viscosity*
