# Supplementary material for: Genome-wide association study for kernel composition and flour pasting behavior in wholemeal maize flour
Source: BMC Plant Biol. 2019 Apr 2;19:123. doi: 10.1186/s12870-019-1729-7 (PMC6444869; doi:10.1186/s12870-019-1729-7)
Supplement: Supplementary file 2 — Table S2. Estimated genetic correlations among quality traits measured in wholemeal flour of a collection of 132 maize inbred lines. In Table S2 one can find the estimated pairwise genetic correlation between the 11 quality trait evaluated for each growing season. (DOCX 22 kb) [file 12870_2019_1729_MOESM2_ESM.docx]

*Additional file 2*

**Table S2. Estimated genetic correlations among quality traits measured in wholemeal flour of a collection of 132 maize inbred lines.**

|  | PR | FI | FT | STL | SIZEL | PV | TV | FV | BD_SqRt | SB1 | SB2 |
| --- | --- | --- | --- | --- | --- | --- | --- | --- | --- | --- | --- |
| PR | - | **0.802** | –0.075 | –0.558 | 0.400 | –0.281 | –0.007 | –0.051 | –0.435 | –0.063 | 0.264 |
| FI | **0.855** | - | 0.356 | –0.664 | 0.669 | –0.416 | –0.110 | –0.129 | –0.571 | –0.118 | 0.307 |
| FT | –0.051 | 0.368 | - | –0.335 | 0.491 | –0.287 | –0.173 | –0.168 | –0.286 | –0.137 | 0.125 |
| STL | –0.298 | –0.438 | –0.367 | - | –0.487 | 0.375 | 0.229 | 0.278 | 0.383 | 0.244 | –0.074 |
| SIZEL | 0.537 | 0.717 | 0.456 | –0.297 | - | –0.402 | –0.060 | –0.136 | –0.545 | –0.162 | 0.247 |
| PV | –0.307 | –0.385 | –0.142 | 0.362 | –0.272 | - | **0.751** | 0.690 | **0.924** | 0.616 | 0.071 |
| TV | 0.008 | 0.033 | –0.029 | 0.113 | 0.122 | **0.759** | - | 0.665 | 0.531 | 0.517 | 0.320 |
| FV | –0.086 | –0.086 | 0.036 | 0.258 | –0.096 | 0.669 | **0.747** | - | 0.527 | **0.808** | 0.507 |
| BD_SqRt | –0.415 | –0.576 | –0.246 | 0.454 | –0.508 | **0.852** | 0.395 | 0.504 | - | 0.554 | –0.139 |
| SB1 | –0.187 | –0.202 | –0.040 | 0.351 | –0.310 | 0.562 | 0.535 | **0.950** | 0.473 | - | 0.593 |
| SB2 | 0.148 | 0.276 | 0.128 | 0.011 | 0.132 | –0.101 | 0.266 | 0.524 | –0.229 | 0.616 | - |

*The genetic correlations were calculated independently for each growing season (year 2011 and year 2012). Values above the diagonal correspond to the genetic correlations among quality traits measured in the first growing season (2011); values below the diagonal correspond to the genetic correlations among quality traits measured in the second growing season (2012). In bold are highlighted the strong genetic correlations (|r| > 0.8)*

*Quality traits: PR –percentage of protein; FI – percentage of fiber; FT – percentage of fat; STL – percentage of starch in lyophilized flour; SIZEL – mean particle size in lyophilized flour; PV – peak (maximum) viscosity; TV – trough (minimum) viscosity; FV – final viscosity; BD_SqRt – squared-root transformed values of the breakdown viscosity; SB1 – setback from trough viscosity; SB2 – setback from peak viscosity*
