# Supplementary material for: Genome-wide association study for kernel composition and flour pasting behavior in wholemeal maize flour
Source: BMC Plant Biol. 2019 Apr 2;19:123. doi: 10.1186/s12870-019-1729-7 (PMC6444869; doi:10.1186/s12870-019-1729-7)
Supplement: Supplementary file 3 — Table S3. Phenotypic values (range, and mean ± standard deviation) for 11 quality traits measured in 132 maize inbred lines. In Table S3 one can find for the 11 quality trait evaluated the summary statistics on phenotypic data for each growing season and across growing seasons. (DOCX 24 kb) [file 12870_2019_1729_MOESM3_ESM.docx]

*Additional file 3*

**Table S3. Phenotypic values (range, and mean ± standard deviation) for 11 quality traits measured in 132 maize inbred lines.**

|  | Range (minimum – maximum) | | Mean ± standard deviation | | |
| --- | --- | --- | --- | --- | --- |
| Trait abbreviation ^1^ | 2011 | 2012 | 2011 | 2012 | AGS ^2^ |
| PR | 9.72 – 15.76 | 9.12 – 15.04 | 12.73 ± 1.09 | 12.07 ± 1.13 | 12.45 ± 1.15 |
| FI | 1.79 – 2.92 | 1.70 – 2.80 | 2.32 ± 0.21 | 2.29 ± 0.22 | 2.31 ± 0.21 |
| FT | 4.02 – 5.19 | 4.30 – 5.39 | 4.61 ± 0.21 | 4.78 ± 0.22 | 4.69 ± 0.23 |
| STL ^3^ | 64.83 – 76.04 | 61.47 – 73.43 | 69.96 ± 2.17 | 67.57 ± 2.59 | 68.89 ± 2.65 |
| SIZEL ^3^ | 82.72 – 210.71 | 108.47 – 212.11 | 144.78 ± 26.17 | 164.22 ± 22.61 | 153.30 ± 26.47 |
| PV | 8.37E2 – 5.30E3 | 5.51E2 – 5.93E3 | 3.10E3 ± 9.80E2 | 3.06E3 ± 1.15E3 | 3.09E3 ± 1.06E3 |
| TV | 6.37E2 – 3.42E3 | 8.30E2 – 3.49E3 | 1.98E3 ± 5.44E2 | 2.09E3 ± 5.04E2 | 2.03E3 ± 5.29E2 |
| FV | 3.01E3 – 9.04E3 | 3.06E3 – 9.76E3 | 6.12E3 ± 1.19E3 | 6.29E3 ± 1.36E3 | 6.20E3 ± 1.27E3 |
| BD_SqRt ^4^ | 3.32 – 56.60 | 1.00 – 56.08 | 31.86 ± 11.18 | 27.74 ± 14.67 | 30.05 ± 12.98 |
| SB1 | 1.57E3 – 6.55E3 | 2.01E3 – 6.71E3 | 4.04E3 ± 9.33E2 | 4.16E3 ± 1.02E3 | 4.09E3 ± 9.71E2 |
| SB2 | 5.38E2 – 4.84E3 | 9.38E2 – 5.26E3 | 2.88E3 ± 8.95E2 | 3.10E3 ± 9.07E2 | 2.97E3 ± 9.05E2 |

*Eleven quality traits were measured in wholemeal maize flour from each of the 132 maize inbred lines evaluated in two growing seasons (2011 and 2012)*

*^1^ Traits: PR* – *Protein, in %; FI* – *Fiber, in %; FT* – *Fat, in %; STL* – *Starch, in %; SIZEL* – *Mean particle size, in µm; PV* – *Peak (maximum) viscosity, in cP; TV* – *Trough (minimum) viscosity, in cP; FV* – *Final viscosity, in cP; BD_SqRt* – *Breakdown, in cP; SB1* – *Setback from trough viscosity, in cP; SB2* – *Setback from peak viscosity, in cP*

*^2^ AGS stands for across growing seasons*

*^3^ Traits values obtained from lyophilized flour*

*^4^ Breakdown values were squared-root transformed*
