# Supplementary material for: Genome-wide association study for kernel composition and flour pasting behavior in wholemeal maize flour
Source: BMC Plant Biol. 2019 Apr 2;19:123. doi: 10.1186/s12870-019-1729-7 (PMC6444869; doi:10.1186/s12870-019-1729-7)
Supplement: Supplementary file 4 — Table S4. Observed inflation factors for the models tested in genome-wide association (GWAS) analysis. In Table S4 one can find the inflation factors for each of the three different models that were tested to detect significant marker-trait associations: the naïve model, a model accounting for population structure; and a model accounting for familiar relatedness. (DOCX 21 kb) [file 12870_2019_1729_MOESM4_ESM.docx]

*Additional file 4*

**Table S4. Observed inflation factors for the models tested in genome-wide association (GWAS) analysis.**

| # | Trait | Naïve | Eigen | Adaptive Kinship ^1^ |
| --- | --- | --- | --- | --- |
|  |  |  |  |  |
| 1 | Protein content (PR) | 1.409 | 1.133 | 1.062 |
| 2 | Fiber content (FI) | 1.730 | 1.080 | 1.079 |
| 3 | Fat content (FT) | 1.578 | 1.055 | 1.033 |
| 4 | Starch content (STL) | 1.658 | 1.125 | 1.091 |
| 5 | Mean particle size (SIZEL) | 1.647 | 1.105 | 1.108 |
| 6 | Peak viscosity (PV) | 1.690 | 1.077 | 1.048 |
| 7 | Trough viscosity (TV) | 1.270 | 1.064 | 1.019 |
| 8 | Final viscosity (FV) | 1.246 | 1.051 | 1.036 |
| 9 | Breakdown viscosity (BD_SqRt) | 1.782 | 1.098 | 1.090 |
| 10 | Setback from trough viscosity (SB1) | 1.138 | 1.038 | 1.038 |
| 11 | Setback from peak viscosity (SB2) | 1.149 | 1.053 | 1.036 |

*^1^ Calculated according to Listgarten et al., 2012; Rincent et al., 2014. Inflation factor for the adaptive kinship model corresponds to the average value across chromosomes.*
