# Supplementary material for: Genome-wide association study for kernel composition and flour pasting behavior in wholemeal maize flour
Source: BMC Plant Biol. 2019 Apr 2;19:123. doi: 10.1186/s12870-019-1729-7 (PMC6444869; doi:10.1186/s12870-019-1729-7)
Supplement: Supplementary file 6 — Table S6. Percentage of associated SNPs with opposite effects on each traits value, and the range of phenotypic variance explained. In Additional file 6: Table S6 one can find the summarized information on the overall abundance of alleles increasing and decreasing the trait value in the inbred line collection; information on the range of phenotypic variance explained by significantly associated SNPs; information on the effect of rare allele explaining the largest phenotypic variance. (DOCX 23 kb) [file 12870_2019_1729_MOESM6_ESM.docx]

*Additional file 6*

**Table S6. Percentage of associated SNPs with opposite effects on each traits value, and the range of phenotypic variance explained.**

| Trait | %SNPs _DECREASE_ | %SNPs _INCREASE_ | V_MIN_  (SNP ID; location) | V_MAX_  (SNP ID; location) | SNP_EFFECT_ |
| --- | --- | --- | --- | --- | --- |
| Protein (PR), in % | 91.67 | 8.33 | 0.066  (rs131210797; chr1: 12,944,506 bp) | 0.099  (rs131232105; chr1: 32,313,522 bp) | (−)0.56% |
| Fiber (FI), in % | 93.75 | 6.25 | 0.065  (rs131178018, rs131180681, rs131180682, rs131180679; chr1: 267,638,749-267,677,992 bp) | 0.090  (rs132587158; chr10: 118,706,425 bp) | (−)0.09% |
| Fat (FT), in % | 40.00 | 60.00 | 0.079  (rs131178929; chr4: 235,742,784 bp) | 0.102  (rs132129576; chr4: 118,410,565 bp) | (+)0.12% |
| Starch (STL), in % | 57.14 | 42.86 | 0.068  (rs128781599; chr1: 158,498,846 bp) | 0.096  (rs131186983; chr1: 23,242,656 bp) | (−)1.02% |
| Mean particle size (SIZEL), in μm | 33.33 | 66.67 | 0.105  (rs131176855; chr6: 114,921,724 bp) | 0.148  (rs131635762; chr7: 101,287,298 bp) | (−)18.13 μm |
| Peak viscosity (PV), in cP | 33.33 | 66.67 | 0.080  (rs128531960; chr10: 60,163,242 bp) | 0.115  (rs131765763; chr10: 60,163,343 bp) | (−)383.98 cP |
| Trough viscosity (TV), in cP | 100.00 | 0.00 | 0.094  (rs130376196; chr6: 116,511,199 bp) | 0.118  (rs129057461; chr2: 44,381,952 bp) | (−)184.4 cP |
| Final viscosity (FV), in cP | 50.00 | 50.00 | 0.096  (rs130828069; chr8: 102,473,765 bp) | 0.111  (rs131180967; chr3: 173,420,781 bp) | (−)422.1 cP |
| Breakdown viscosity (BD_SqRt), in cP | 45.45 | 54.55 | 0.073  (rs131664622; chr8: 26,757,485 bp) | 0.112  (rs131765763; chr10: 60,163,343 bp) | (−)4.82 cP |
| Setback from trough viscosity (SB1), in cP | 50.00 | 50.00 | 0.111  (rs131176534; chr6: 35,091,373 bp) | 0.113  (rs131180967; chr3: 173,420,781 bp) | (−)298.93 cP |
| Setback from peak viscosity (SB2), in cP | 100.00 | 0.00 | 0.133  (rs131182722, rs131182721, rs132138984; chr4: 141,121,126-141,298,543 bp) | 0.156  (rs132163268; chr4: 174,747,548 bp) | (−)503.09 cP |

*%SNPs_DECREASE_ – Percentage of associated SNPs for which the effect of the rare allele resulted in a decrease of the trait value when compared with inbred lines carrying the most frequent allele; %SNPs_INCREASE_ - Percentage of associated SNPs for which the effect of the rare allele results in an increase of the trait value when compared with the inbred lines carrying the most frequent allele; V_MIN_ – smallest proportion of variance explained by a significant SNP; V_MAX_ – largest proportion of variance explained by a significant SNP; SNP_EFFECT -_ Effect of the SNP variant explaining the largest proportion of the trait phenotypic variance*
