# Supplementary material for: Genome-wide association study for kernel composition and flour pasting behavior in wholemeal maize flour
Source: BMC Plant Biol. 2019 Apr 2;19:123. doi: 10.1186/s12870-019-1729-7 (PMC6444869; doi:10.1186/s12870-019-1729-7)
Supplement: Supplementary file 8 — Table S8. Maize inbred lines with available quality data, known pedigree, kernel color, and endosperm type. In Additional file 8: Table S8 one can find the list of 132 inbred lines that yielded sufficient kernels to proceed for quality analysis. The underlined inbred lines correspond to the lines derived entirely from Portuguese traditional maize populations, according to the Portuguese Plant Germplasm Bank records. (DOCX 34 kb) [file 12870_2019_1729_MOESM8_ESM.docx]

*Additional file 8*

**Table S8. Maize inbred lines with available quality data, known pedigree, kernel color, and endosperm type.**

| # | Inbred line | Collection code | Recorded pedigree | Kernel color | Endosperm type |
| --- | --- | --- | --- | --- | --- |
| 1 | P147/83 PB367 38-11/2 Fasciada | L036 | 38 - 11/2 | white | dent |
| 2 | P149/83 PB366 38-11/1 Fasciada | L037 | 38 - 11/1 | white | dent |
| 3 | P95/83 PB363 WF9W (C.B.) Fasciada | L042 | WF9W (C.B.) | white | dent |
| 4 | PB100 | L043 | Branco Vila Nova de Ourém | white | flint |
| 5 | PB108TR | L048 | American white ﬂint | white | flint |
| 6 | PB109 | L049 | Neb.505W | white | dent |
| 7 | PB111 | L051 | Verdeal de Bastos | white | flint |
| 8 | PB111(TR) | L052 | Verdeal de Bastos | white | flint |
| 9 | PB117 | L054 | (PB 40 x 38-11) PB 40 | white | flint |
| 10 | PB135 | L057 | Unha de Porco | white | flint |
| 11 | PB18 | L059 | Pombeiro | white | flint |
| 12 | PB202 | L065 | S401W | white | dent |
| 13 | PB255 | L087 | S302W | white | intermediate |
| 14 | PB256 | L088 | (PB52 x KY49) PB52 | white | flint |
| 15 | PB257 | L089 | (S5C1 x PB256) PB256(2) | white | flint |
| 16 | PB258 | L090 | (PB117 x S146) PB117(2) | white | intermediate |
| 17 | PB260 | L092 | (PB6 x PB7) PB6(2) | white | flint |
| 18 | PB261 | L093 | (PB40 x PB75) PB40 | white | flint |
| 19 | PB265 | L094 | (S158 x PB264) PB264 | white | dent |
| 20 | PB266 | L095 | (WF9 x PB53) WF9 | white | dent |
| 21 | PB266 +Fasciada | L096 | (WF9 x PB53) WF9 | white | dent |
| 22 | PB31 | L109 | Unha de Porco | white | flint |
| 23 | PB365 | L114 | *NA* | white | dent |
| 24 | PB369 | L115 | A166 | white | dent |
| 25 | PB372 | L116 | AS-Y | white | dent |
| 26 | PB38 | L122 | Arrancada | white | flint |
| 27 | PB4 | L123 | Rustler | white | flint |
| 28 | PB40 | L124 | Belga | white | flint |
| 29 | PB54 | L126 | Cem Dias Grande | white | flint |
| 30 | PB56 | L128 | Neb.505W | white | dent |
| 31 | PB98(TR) | L146 | Regional de Fafe | white | flint |
| 32 | PP11 | L147 | Cem dias | white | flint |
| 33 | PP85R | L149 | Dente de Cavalo de Gaia | white | intermediate |
| 34 | PP9 | L150 | Gigante da Feira | white | flint |
| 35 | PP91 | L151 | Wisconsin gigante | white | dent |
| 36 | PV135 | L155 | Unha de Porco | white | flint |
| 37 | 3022 | L009 | Mistoral Variety | yellow | dent |
| 38 | B14A | L017 | (Iowa Stiff Stalk Synthetic) Cuzco x B14 | yellow | dent |
| 39 | C106 | L021 | (LDG(K) x L317 | yellow | dent |
| 40 | CI31A | L022 | Midland “A” O.P. | yellow | intermediate |
| 41 | F542 | L023 | F47 x B14 | yellow | dent |
| 42 | F568 | L025 | *NA* | yellow | dent |
| 43 | L289 | L030 | O. P. Lancaster | yellow | dent |
| 44 | Oh51A | L035 | (Oh51 x Oh17) Oh51 | yellow | dent |
| 45 | P8 | L041 | O. P. Reid Yellow Dent (Polint.) | yellow | dent |
| 46 | PB102 | L045 | MBG7 x 14 | yellow | dent |
| 47 | PB105 | L047 | MBG33 x 25 | yellow | flint |
| 48 | PB194 | L062 | S160 | yellow | dent |
| 49 | PB199 | L064 | S28C1 | yellow | intermediate |
| 50 | PB21 | L068 | S366 | yellow | dent |
| 51 | PB213 | L069 | S158 (Wx) | yellow | dent |
| 52 | PB216 | L072 | S1C5 | yellow | dent |
| 53 | PB217 | L073 | S1C7 | yellow | dent |
| 54 | PB229 | L075 | S23B3 | yellow | dent |
| 55 | PB234 | L077 | S24C4 | yellow | dent |
| 56 | PB236 | L078 | S24C6 | yellow | dent |
| 57 | PB237 | L079 | S24C6 | yellow | dent |
| 58 | PB244 | L081 | S28BL | yellow | dent |
| 59 | PB246 | L082 | S28B3 | yellow | dent |
| 60 | PB250 | L084 | S37C2 | yellow | dent |
| 61 | PB253 | L086 | S47A | yellow | dent |
| 62 | PB273 | L101 | 2006 x 2031(E.A) | yellow | flint |
| 63 | PB375 | L119 | Nº494-Vilariça-Moncorvo (S.R.) | yellow | flint |
| 64 | PB68 | L133 | (S1460 x Min.13) Min.13(2) | yellow | dent |
| 65 | PB89 | L143 | (S1460 x PB89) PB89(3) | yellow | intermediate |
| 66 | PB93 | L144 | S366 | yellow | dent |
| 67 | R59 | L156 | L313 x ILL.Low Ear | yellow | intermediate |
| 68 | W22 | L160 | ILLB10 x W25 | yellow | dent |
| 69 | WF9 | L163 | Wilson Farm Reid | yellow | dent |
| 70 | 95 | L001 | Lancaster Surcrop | yellow-orange | dent |
| 71 | 2033 | L003 | Isseu de M 14 | yellow-orange | dent |
| 72 | 2440 | L004 | B14 x Urdos O. P. | yellow-orange | dent |
| 73 | 2579 | L006 | O. P. Wisconsin | yellow-orange | dent |
| 74 | 3040 | L011 | C103 x T8 | yellow-orange | dent |
| 75 | A619 | L014 | (A171 x Oh43) Oh43(3) | yellow-orange | dent |
| 76 | B37 | L018 | Iowa Stiff Stalk Synthetic | yellow-orange | dent |
| 77 | B49 | L019 | A277 x 41-2504B | yellow-orange | dent |
| 78 | FR632 | L027 | *NA* | yellow-orange | dent |
| 79 | FR9 | L028 | Reid Yellow Dent | yellow-orange | dent |
| 80 | I45B | L029 | (Oh40B x W8) (CR) | yellow-orange | dent |
| 81 | Oh43 | L034 | Oh40B x W8 | yellow-orange | dent |
| 82 | P570/88 Fasciada | L039 | Variedade Regional Ramosa | yellow-orange | intermediate |
| 83 | P572/88 Fasciada | L040 | Variedade Regional Ramosa | yellow-orange | flint |
| 84 | PB104 | L046 | MBG 7 x 14 | yellow-orange | flint |
| 85 | PB110 | L050 | MBG 7 x 14 | yellow-orange | dent |
| 86 | PB116 | L053 | MBG 7 x 14 | yellow-orange | flint |
| 87 | PB118 | L055 | MBG 7 x 14 | yellow-orange | dent |
| 88 | PB14 | L058 | Torre de Quintela | yellow-orange | flint |
| 89 | PB192 | L061 | Escuro de Elvas | yellow-orange | flint |
| 90 | PB214 | L070 | S1C1 | yellow-orange | dent |
| 91 | PB215 | L071 | S1C5 | yellow-orange | flint |
| 92 | PB219 | L074 | S1C7 | yellow-orange | dent |
| 93 | PB240 | L080 | S25C3 | yellow-orange | dent |
| 94 | PB251 | L085 | S47A | yellow-orange | dent |
| 95 | PB269 | L097 | Hembrilla x FR43 | yellow-orange | flint |
| 96 | PB270 | L098 | A344(TR) x T20C(TR) | yellow-orange | dent |
| 97 | PB271 | L099 | Hembrilla x FR43 | yellow-orange | flint |
| 98 | PB272 | L100 | (PB60 x U.S.A.) PB60 | yellow-orange | flint |
| 99 | PB275 | L102 | A344(TR) x T20(TR) | yellow-orange | dent |
| 100 | PB297 | L103 | Sint. Amarelo Dent | yellow-orange | dent |
| 101 | PB301 | L104 | Sint. Amarelo Dent | yellow-orange | flint |
| 102 | PB304 | L105 | Regional do Carreço | yellow-orange | flint |
| 103 | PB309 | L108 | E.P.S. 1 | yellow-orange | flint |
| 104 | PB311 | L110 | E.P.S. 2 | yellow-orange | intermediate |
| 105 | PB312 | L111 | E.P.S. 2 | yellow-orange | flint |
| 106 | PB317 | L113 | (PB190 x PB92) PB190(2) | yellow-orange | flint |
| 107 | PB374 | L118 | Nº517-Resende-Viseu (S.R.) | yellow-orange | flint |
| 108 | PB79 | L137 | Nostrano dell' Isola | yellow-orange | flint |
| 109 | PT12 | L152 | Zorrinho M42 | yellow-orange | flint |
| 110 | PT14 | L153 | Robym (Pothechefstroom) | yellow-orange | flint |
| 111 | T127(TR) | L158 | *NA* | yellow-orange | dent |
| 112 | TR | L159 | Troyer Reid | yellow-orange | dent |
| 113 | W64A | L162 | WF9 x CI.187-2 (CR) | yellow-orange | intermediate |
| 114 | 2594 | L007 | WM13 x EP1 | orange | flint |
| 115 | 2612 | L008 | 38-11 x W182B | orange | dent |
| 116 | 3039 | L010 | (C103 x T8) T8 | orange | flint |
| 117 | PB190 | L060 | Escuro de Elvas | orange | flint |
| 118 | PB207 | L067 | Rojo Viñoso de Aragón | orange | intermediate |
| 119 | PB305 | L106 | Regional do Carreço (S.R.) | orange | flint |
| 120 | PB307 | L107 | E.P.S. 1 | orange | flint |
| 121 | PB315 | L112 | Sint. Amarelo Flint | orange | flint |
| 122 | PB377 | L120 | Nº388-Silves (S.R.) | orange | flint |
| 123 | PB378 | L121 | Santa Catarina (Tavira) S.R | orange | flint |
| 124 | PB57A | L129 | PB57 - S.Pancrásio | orange | flint |
| 125 | PB60 | L131 | Nostrano dell' Isola | orange | flint |
| 126 | PB79/2 | L138 | Nostrano dell’Isola | orange | flint |
| 127 | PB80 | L139 | S. Pancrázio | orange | flint |
| 128 | PB86(TR) | L141 | Torre de Quintela | orange | flint |
| 129 | PB86/1 | L142 | Torre de Quintela | orange | flint |
| 130 | PT2 | L154 | Gigante M83 | orange | flint |
| 131 | WR3 | L164 | Reid Yellow Dent | orange | flint |
| 132 | PB71/1 | L135 | (S1460 x PB71) PB71 | red | dent |

### *The underlined inbred lines are derived entirely from traditional Portuguese maize populations (according to the Portuguese Plant Germplasm Bank records)*

### *NA stands for information not available*
