# Supplementary material for: Genome-wide association study for kernel composition and flour pasting behavior in wholemeal maize flour
Source: BMC Plant Biol. 2019 Apr 2;19:123. doi: 10.1186/s12870-019-1729-7 (PMC6444869; doi:10.1186/s12870-019-1729-7)
Supplement: Supplementary file 9 — Table S9. Number of maize inbred lines grouped accordingly to their kernel color and endosperm type. In Additional file 9: Table S9 one can find the summary of the number of inbred lines with the different kernel colors and endosperm types. (DOCX 21 kb) [file 12870_2019_1729_MOESM9_ESM.docx]

*Additional file 9*

Table 2. Number of maize inbred lines grouped accordingly to their kernel color and endosperm type.

| Kernel color | Endosperm type | | | |
| --- | --- | --- | --- | --- |
|  | Flint | Intermediate | Dent | Total |
| White | 20 | 3 | 13 | 36 |
| Yellow | 3 | 4 | 26 | 33 |
| Yellow-orange | 18 | 3 | 23 | 44 |
| Orange | 16 | 1 | 1 | 18 |
| Red | − | − | 1 | 1 |
| Total | 57 | 11 | 64 | 132 |
